# Asymmetric base-pair opening drives helicase unwinding dynamics

**DOI:** 10.1073/pnas.1901086116

**Published:** 2019-10-18

**Authors:** Francesco Colizzi, Cibran Perez-Gonzalez, Remi Fritzen, Yaakov Levy, Malcolm F. White, J. Carlos Penedo, Giovanni Bussi

**Affiliations:** ^a^Molecular and Statistical Biophysics, Scuola Internazionale Superiore di Studi Avanzati, 34136 Trieste, Italy;; ^b^Computational Biology Node, Institute for Research in Biomedicine (IRB Barcelona), The Barcelona Institute of Science and Technology, 08028 Barcelona, Spain;; ^c^School of Biology, Biomedical Sciences Research Complex, University of St. Andrews, St. Andrews KY16 9ST, United Kingdom;; ^d^Scottish Universities Physics Alliance School of Physics and Astronomy, University of St. Andrews, St. Andrews KY16 9SS, United Kingdom;; ^e^Department of Structural Biology, Weizmann Institute of Science, Rehovot 7610001, Israel

**Keywords:** double helix, nucleic acids, simulations, experiments, unwindability

## Abstract

The opening of a Watson–Crick double helix is required for crucial cellular processes, including replication, repair, and transcription. It has long been assumed that RNA or DNA base pairs are broken by the concerted symmetric movement of complementary nucleobases. By analyzing thousands of base-pair opening and closing events from molecular simulations, here, we uncover a systematic stepwise process driven by the asymmetric flipping-out probability of paired nucleobases. We demonstrate experimentally that such asymmetry strongly biases the unwinding efficiency of DNA helicases toward substrates that bear highly dynamic nucleobases, such as pyrimidines, on the displaced strand. Duplex substrates with identical thermodynamic stability are thus shown to be more easily unwound from one side than the other, in a quantifiable and predictable manner. Our results indicate a possible layer of gene regulation coded in the direction-dependent unwindability of the double helix.

## Significance

Six decades after DNA structure was first revealed, fundamental questions remain open. How is the entwined embrace of double-stranded nucleic acids formed or disrupted? How does the energetics underlying this process influence nucleic-acid processing machineries? By combining simulations and experiments, our work addresses these questions and reveals that asymmetric base-pair dynamics drives the stepwise separation of nucleic acid duplexes, predicts the unwinding efficiency of helicases, and intimately relates the intrinsic dynamics of base pairs to the enzymatic mechanism evolved for their opening. Taken together, our data suggest a layer of regulation of the genetic material encoded in the “unwindability” of the double helix.

Since its discovery, the structure of the DNA double helix (1, 2), with its striking implications for DNA replication and DNA recombination (3), has provided a means to probe and understand the molecular biology of “genetic material” (4). Soon after the Watson–Crick (W-C) model was proposed, it was recognized that DNA strand separation was critical to DNA function (5), thus motivating the quest for conditions that would disrupt the W-C hydrogen bonds so as to separate the 2 strands of the DNA double helix (3). However, the way the elementary steps of these conformational transitions affect nucleic-acid processing machineries is still not fully understood.

The properties of DNA and RNA double helices have shaped the structure and mechanisms of proteins endowed with the ability of opening base pairs to perform chemical modification (e.g., methyltransferases) or to allow DNA and RNA remodeling (e.g., helicases). Chemical modifications are usually achieved by the specific flipping out, also named extrusion, of the target nucleobase (6, 7). These types of base-flipping processes have been the focus of intense computational work (8, 9), and NMR studies have further shown the sequence dependence of the flipping rate of internal base pairs in relevant biological contexts (10, 11). More generally, local thermal fluctuations of base pairs (12–15) can transiently separate double-stranded (ds) nucleic acids, and this phenomenon affects the binding, assembly, and translocation of gene-expression machines (16–18). In this respect, A·T- or A·U-rich segments show increased breathing (decreased stability) than G·C-rich segments (14, 15) and are, for instance, more readily unwound by helicases—ATP-fueled motor proteins capable of separating the duplex (16) with a sequence-dependent stepping velocity (19) and processivity (20).

Complementary to base extrusion, the iteration of base-pair opening events at the junction between single-stranded (ss) and ds nucleic acids, also referred to as unzipping, complies with a different set of steric and torsional constraints and approximates the biological process of helix opening by helicases, as suggested by X-ray crystallography (16, 21) and single-molecule experiments (19, 22, 23). The separation of ds nucleic acids is assumed to follow the classical zipper model (24), where base-pair opening occurs as a concerted process with an equivalence (or symmetry) between 2 complementary nucleobases. This assumption has been largely based on the difficulty to observe and characterize the “invisible” intermediate states—for instance, those ss/ds junctions with only 1 of the 2 nucleobases flipped out (25–27)—which are too few and whose duration is too short for experimental determination. In this context, computer simulations could bring about a major productivity leap, providing thermodynamic and kinetic information on transition intermediates that might escape spectroscopic detection. Indeed, the large amount of molecular dynamics (MD) work on the melting of base pairs in various conditions (13, 26–37) suggests that base-pair opening is an asynchronous process, with one base unstacking significantly before the other. However, systematic studies on how the nucleic acid sequence can affect base-pair opening intermediates during the unzipping of ss/ds junctions are lacking. Furthermore, according to the classical zipper model, a G·C repeat, for example, 5′-G_n_-3′/3′-C_n_-5′, will be equally unzipped by a helicase regardless of whether the helicase tracks on the 5′-G_n_-3′ or 5′-C_n_-3′ strand. This is a paradigm that has long influenced the understanding of the nucleic-acid processing apparatus. However, the evidence that base-pair opening can be asynchronous and that the intrinsic dynamics of nucleic acids, even at the single base-pair level, can modulate the function of the genetic material (17–19, 38, 39) posits the challenge of questioning how the mechanism of duplex separation, resolved with atomistic spatiotemporal resolution, can impact the function of nucleic-acid processing machineries.

Here, by combining simulations and experiments, we study how RNA and DNA duplexes unwind and how the energetics underlying the elementary step of base-pair opening influences the function of helicases. First, we present a high-throughput structure-based MD approach to provide a comprehensive view of the unwinding mechanism of double helices. By analyzing thousands of base-pair opening and closing events, we uncover a systematic asymmetry in the dynamics of a W-C base pair that makes 1 of the 2 nucleobases more likely to flip out. Such asymmetric dynamics is encoded in the sequence and affects the intermediate states populated by dsRNA and dsDNA during opening. As a result, base unpairing is, systematically, a stepwise asymmetric process with 1 of the 2 nucleobases being the weak point from which the base pair breaks. Based on this observation, we then explore the impact of asymmetric nucleobase dynamics in the context of helicase unwinding, whereby we predict enhanced unwinding efficiency for pyrimidine-rich substrates on the displaced strand. Finally, using biochemical and fluorescence-based assays, we test our hypothesis by measuring the unwinding of various designed substrates by helicase enzymes. The experiments confirm the predictions and corroborate a model that intimately relates the asymmetric dynamics of base pairs to the unwinding mechanism of helicases. Our results demonstrate that one duplex portion can be more easily unwound from one side than from the other. Taken together, our data suggest a layer of gene regulation encoded in the direction-dependent “unwindability” of the double helix.

## Results

### Extensive Simulations Show Different Pathways of Base-Pair Opening in dsDNA and dsRNA.

**Fig. 1.**
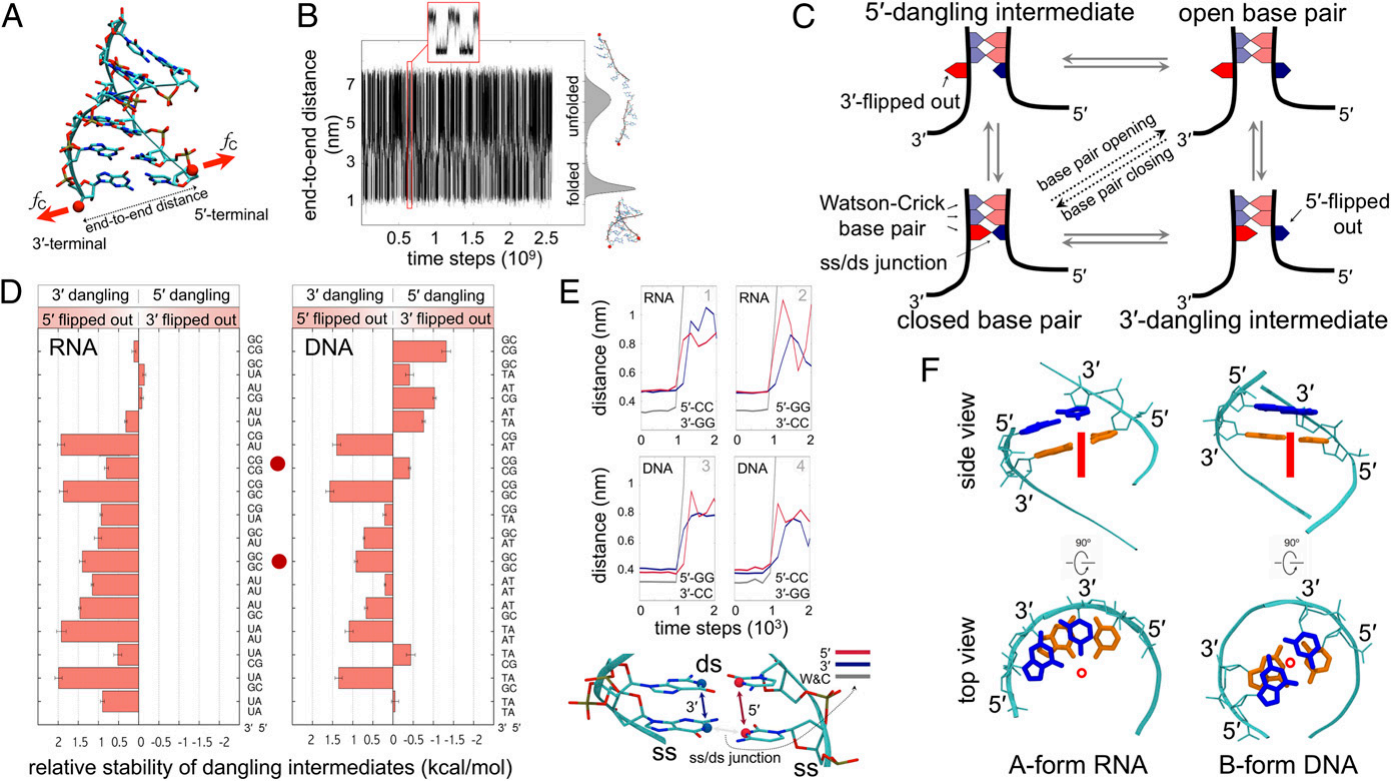
Modeling the formation and rupture of double helices by mimicking constant-force optical-tweezer experiments (59). (*A*) Ribbon molecular graphics representation of the ds nucleic acid model and schematic of the reaction coordinate. Red spheres show the 3′ and 5′ hydroxyl groups of the terminal base pair, defining the end-to-end distance, where the external constant force, *f*_C_, was applied (red arrows; *Methods*). (*B*) Time series of the hopping between folded and unfolded states, zoomed in the red inset. Histogram of the end-to-end distance is on the right, together with sample conformations. (*C*) Schematic of the stepwise mechanism of base-pair opening/closing. The bases in the ss portion are not shown for sake of clarity. (*D*) Free-energy difference between the unbiased population of 5′- and 3′-dangling intermediates (*SI Appendix*, *Methods*) for the base pair at the bottom of each nearest-neighbor combination shown on the vertical axis. Positive values correspond to higher population of 3′-dangling intermediates. Red dots highlight the base-pair combinations explicitly discussed in the main text. Bars indicate SE from bootstrapping (60). (*E*) Time evolution of the base-pair opening process at a ss/ds junction in RNA and DNA. The distances used to detect W-C pairing (gray), 5′-stacking (red), and 3′-stacking (blue) of the closing base pair are shown. Data are averaged over windows of 300 time steps. (*F*) Side and top views of adjacent W-C base pairs in RNA and DNA duplexes. The major axis of the helix is shown as a red circle and a red line in the top and side view, respectively. Sugar-phosphate backbone is in cyan sticks and ribbons. Adjacent base pairs are colored in blue and orange to highlight overlap extension.

By simulating ds separation and annealing in short duplexes, we monitored thousands of base-pair opening and closing events (Fig. 1 *A* and *B*) and systematically analyzed the nucleobase dynamics of the 16 nearest-neighbor base-pair combinations (*Methods* and *SI Appendix*). We observed 2 elementary steps during the opening of each base pair (Fig. 1*C*); hereafter we omit to mention the reverse base-pair closing events, which are observed with statistically identical probabilities in our equilibrium simulation. These steps are: 1) the unpairing and unstacking (also referred to as “flip out”) of a nucleobase, either at the 5′ or 3′ terminus of a ss/ds junction, resulting in a dangling-base intermediate; and 2) the unstacking of the opposite nucleobase, completing the base-pair opening event. We quantified the preference of each nearest-neighbor base-pair combination to follow the pathway passing through either a 3′- or 5′-dangling intermediate (Fig. 1*D*). In other words, we quantified the 5′ or 3′ flip-out events that lead to the opening of a W-C base pair at ss/ds junctions. Two different scenarios emerged when comparing dsRNA to dsDNA (Fig. 1 *D* and *E*).

In RNA duplexes, the nucleobase at the 5′ terminus of a ss/ds junction consistently showed higher propensity to flip out when compared to the complementary W-C base at the 3′ terminus (Fig. 1 *D*, *Left*). That is, the opening of a RNA base pair occurs with higher probability through a 3′-dangling intermediate rather than a 5′-dangling intermediate, thus generating a conserved asymmetry (or directionality) in the opening mechanism. In sharp contrast, in DNA duplexes, both 5′ and 3′ flipped-out intermediates were significantly populated at ss/ds junctions, and their relative population was exclusively modulated by the sequence (Fig. 1 *D*, *Right*). For example, the energetics of unwinding favors the stepwise opening of the ss/ds junction ^5′-NN^CCNN-3′/5′-NNGG^NN-3′^ through the 3′-dangling intermediate ^5′-NNC^CNN-3′/5′-NNGG^NN-3′^ (superscript denotes flipped-out bases) both in RNA and DNA. In the time series plots (Fig. 1*E*), this preference is manifested by the early increment of the stacking distance at the 5′ terminus (red line in panels 1 and 4 of Fig. 1*E*). If the 2 strands are swapped ^5′-NN^GGNN-3′/5′-NNCC^NN-3′^, the preference changes to the 5′-dangling intermediate in DNA ^5′-NN^GGNN-3′/5′-NNC^CNN-3′^, whereas it remains unchanged in RNA (panels 2 and 3 in Fig. 1*E*).

The geometric features of A-type and B-type helices provide a structural interpretation of our results (Fig. 1*F*). In B-DNA, both the 5′ and 3′ ends of a ss/ds junction are equally buried in the helix, and the displacement probability depends only on the sequence. Vice versa, in A-RNA, the bases at the 5′ end of a ss/ds junction are less buried in the neighboring environment, thus facilitating displacement events that lead to 5′ flipped-out intermediates.

The overall dynamics of base-pair opening described here is similar to that observed in accurate atomistic MD simulations (13, 26–37). However, water and ion effects as well as noncanonical structures (13, 30, 37) acting as kinetic traps are by construction omitted from our structure-based model. Nevertheless, these results are consistent with the population of stacked conformers observed in ultrafast spectroscopy experiments on short duplexes (40) and with the analysis of structural databases (25) and can be related to the stabilization provided by dangling ends, although the latter comparison should be interpreted with caution (*SI Appendix*, *Discussion*) (26, 40, 41). Even though the asymmetric intermediate species involved in the opening of a base pair can be difficult to measure experimentally (42), we hypothesized that these intermediates could impact nucleic-acid processing machines. In the next sections, we thus explore the possible consequences of asymmetric base-pair opening on the function of helicases—which, in turn, are a proxy of base-pair dynamics (17–19).

### Relating the Asymmetric Dynamics of Base Pairs to the Unwinding Efficiency of Helicases: The Unwindability Index.

**Fig. 2.**
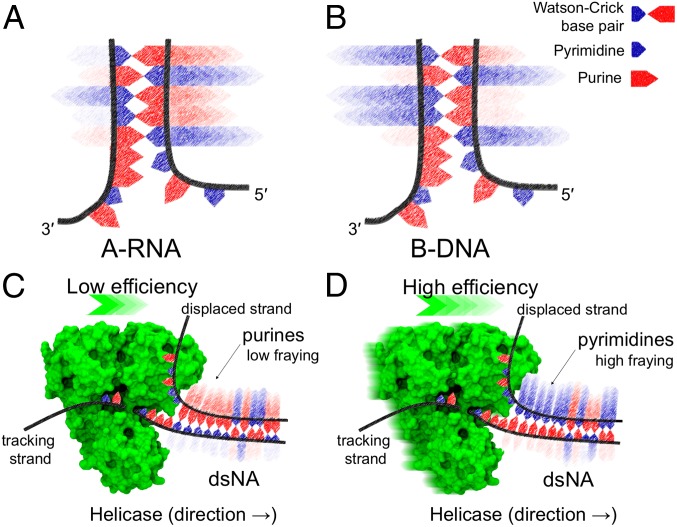
Pictorial representation of nucleic acids unwinding catalyzed by SF1 and SF2 helicases. The flip-out probability of nucleobases at ss/ds junctions is depicted with fading arrows. High color intensity corresponds to a high flip-out probability. (*A*) In A-RNA, the flip out of bases at the 5′-end is consistently favored over the flip out of the complementary base at the 3′-end. Vice versa, in B-DNA (*B*), the direction of base-pair opening depends on the sequence only. We postulate that helicase unwinding efficiency is low when purines are on the displaced strand (*C*) and high when pyrimidines are on the displaced strand (*D*). Helicase structure (NS3; Protein Data Bank ID code 3O8R) (61) rendered with Visual Molecular Dynamics (62).

To examine whether the observed asymmetry influences the function of helicases, we started by analyzing base-pair dynamics in the context of superfamily (SF) 1 and 2 helicases (Fig. 2) (43), monomeric or dimeric nontoroidal enzymes comprising the minimal building-block domains necessary for helicase activity. From a general standpoint, SF1 and SF2 helicases unwind the duplexes by first loading onto an overhanging terminal region and then translocating toward the duplex along this loading strand, thereby peeling off the complementary bases. In particular, the migration of the tracking strand through a specific protein tunnel facilitates, actively or passively, the displacement of the complementary strand by steric exclusion (16, 43).

**Fig. 3.**
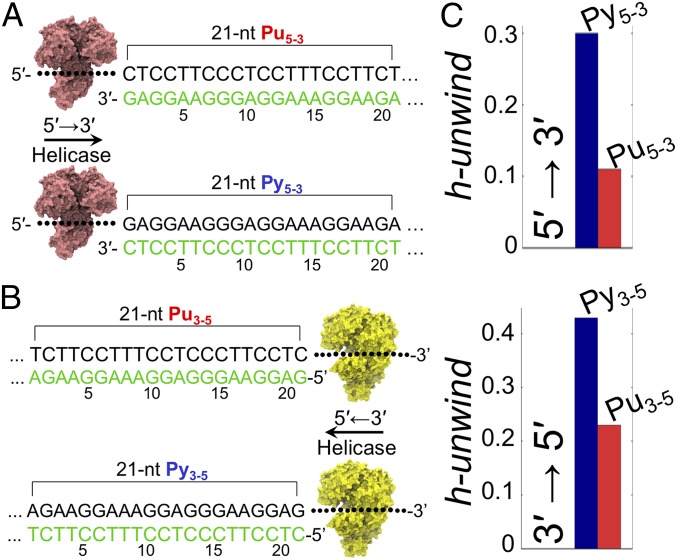
The h-unwind predicts a direction-dependent unwinding efficiency of helicases. (*A*) DNA duplexes Pu_5-3_ and Py_5-3_ have a 21-nt sequence in the displaced strand (in green) that is homopurine and homopyrimidine, respectively. (*B*) Pu_3-5_ and Py_3-5_ have a homopurine and homopyrimidine sequence in the displaced strand (in green), respectively. (*C*) Duplexes with homopyrimidines in the displaced strand (Py_5-3_ and Py_3-5_) have higher h-unwind values and are thus predicted to be more efficiently unwound than the homopurine analogs (Pu_5-3_ and Pu_5-3_).

A key feature of the helicase unwinding model that we propose below is that the population of stacked nucleobases at the displaced strand represents, by direct or indirect interaction with the helicase, a bottleneck for the unwinding activity. Note that the MD simulations presented herein show that purines have lower propensity to flip out than pyrimidines (see, for example, the CC/GG and GG/CC combinations highlighted by a red dot in Fig. 1*D*). In the helicase context, we surmise that the lower flipping propensity of purines at the displaced terminus may result in lower unwinding efficiency. That is, the action of helicases would be impeded by the purines at the displaced strand. In this scenario, duplex opening would thus show a low and high efficiency when purines and pyrimidine are displaced, respectively (Fig. 2 *C* and *D*). To provide a quantitative prediction of unwinding efficiencies, here we heuristically introduce a “helix unwindability” index (“h-unwind”) that allows direct comparison of simulations and experiments (*SI Appendix*, *Methods*). H-unwind is inversely related to the population of stacked nucleobases at the displaced strand—thus, the higher the h-unwind value, the higher the helicase unwinding efficiency. Two sets of DNA constructs were then designed, and the corresponding h-unwind was computed (Fig. 3). The constructs contained a 21-nucleotide (nt) duplex region with the displaced strand consisting entirely of purines (Pu_5-3_ for 5′ → 3′ processing helicases and Pu_3-5_ for 3′ → 5′ helicases) or pyrimidines (Py_5-3_ for 5′ → 3′ helicases and Py_3-5_ for reverse polarity; Fig. 3 *A* and *B*). Although the 2 sets of DNA had the same duplex portion (with swapped strands), the h-unwind allows to differentiate them and predicts Py_5-3_, Py_3-5_ to be more efficiently unwound than Pu_5-3_, Pu_3-5_ by a detectable amount (Fig. 3*C*). We next moved from the in silico predictions to in vitro assays.

### Pyrimidines on the Displaced Strand of DNA Duplexes Facilitate Helicase Unwinding.

**Fig. 4.**
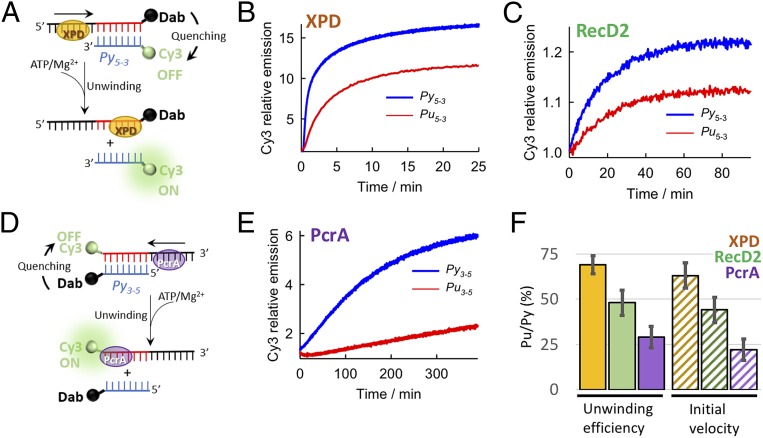
Proof of concept experiments show that duplexes with homopyrimidines on the displaced strand (Py_5-3_, Py_3-5_) are more easily unwound by helicases than the homopurine homologs (Pu_5-3_, Pu_3-5_). Schematic of the fluorescence quenching assay used to investigate duplex unwinding by helicases with 5′ → 3′ (*A*) or 3′ → 5′ (*D*) directionality. ATP addition initiates unwinding and results in strand separation with recovery of Cy3 emission, which is otherwise quenched by Dab in the duplex. Normalized variation in fluorescence intensity of Cy3 as a function of time for Py_5-3_-XPD (blue) and Pu_5-3_-XPD (red) (*B*), Py_5-3_-RecD2 (blue) and Pu_5-3_-RecD2 (red) (*C*), and Py_3-5_-PcrA (blue) and Pu_3-5_-PcrA (red) (*E*). (*F*) Percentage of unwinding amplitude and initial velocity (pattern) observed for homopurine sequences in the displaced strand relative to homopyrimidine sequences for the 3 helicases investigated.

The 2 sets of DNA constructs (Py_5-3_, Pu_5-3_ and Py_3-5_, Pu_3-5_, described in the previous section) were combined with a 21-nt ss overhang and were labeled with indocarbocyanine (Cy3) fluorophore and the 4-([4-(dimethylamino)phenyl]azo)benzoic acid succinimidyl ester (Dab) quencher (44) at duplex termini (Fig. 4 and *SI Appendix*, Table S1 for complete sequences). Before measuring the efficiency of helicase enzymes to unwind such constructs, we carried out melting experiments (*SI Appendix*, Fig. S2) to confirm that both the fluorescence labeling and Pu/Py strand swapping had no impact on duplex thermodynamic stability—a fundamental feature for the aims of the helicase unwinding assays described below.

We then assessed the unwinding activity of 3 DNA helicases, XPD, RecD2, and PcrA (Fig. 4). XPD and RecD2 helicases are members of SF2 and SF1, respectively, and both translocate along DNA with a 5′ → 3′ polarity (43). To probe the general validity of our asymmetric model, we also analyzed the unwinding activity of SF1 helicase PcrA, which moves along DNA in a 3′ → 5′ direction (45).

The XPD helicase unwinds DNA during nucleotide excision repair (46). The unwinding time courses showed a progressive increase in Cy3 emission, indicative of separation of annealed strands (Fig. 4*A*), reaching a plateau in a similar timescale (∼25 min) for both substrates (Fig. 4*B*). However, the relative increase in Cy3 emission, reflecting the amount of ss product formed, was significantly lower for Pu_5-3_, which reached only 64% of the signal observed for Py_5-3_ (Fig. 4*F*). The relative difference in unwinding efficiency between Pu_5-3_ and Py_5-3_ was further confirmed by native polyacrylamide gel electrophoresis (PAGE) using DNA constructs carrying only the Cy3 fluorophore (*SI Appendix*, Fig. S3). Comparable differences in relative unwinding efficiencies were also obtained when using a 10-fold molar excess of a 21-nt unstructured and noncomplementary trap sequence that sequesters excess helicase protein in solution (*SI Appendix*, Fig. S4 *A* and *B*). Similar unwinding efficiencies were observed in the presence of either a 12-nt (*SI Appendix*, Fig. S4*C*) or a 21-nt (*SI Appendix*, Fig. S4*D*) complementary to the Cy3-labeled displaced strand that prevents duplex reannealing. Oligonucleotide trapping sequences are listed in *SI Appendix*, Table S4.

We then used the SF1 helicase RecD2 to unwind Pu_5-3_ and Py_5-3_. RecD2 is a homolog of the RecD subunit of the bacterial RecBCD enzyme involved in dsDNA repair (47). Here, the unwinding activity led to an increase in Cy3 emission until reaching a plateau at ∼60 min (Fig. 4*C*). The fluorescence intensity profiles of Pu_5-3_ unwound by RecD2 exhibited only a 48% recovery of the Cy3 signal level detected for Py_5-3_ (Fig. 4*F*). Native PAGE using only Cy3-labeled constructs confirmed a 2-fold lower unwinding efficiency for Pu_5-3_ compared to Py_5-3_. (*SI Appendix*, Fig. S5). Thus, RecD2 unwinds DNA duplexes containing homopyrimidine sequences (Py_5-3_) in the displaced strand more efficiently than homopurine sequences (Pu_5-3_), and with a slightly more pronounced bias toward homopyrimidine sequences in the displaced strand than XPD (Fig. 4*F*).

Next, we tested the unwinding efficiency of the *Bacillus stearothermophilus* PcrA helicase, which shows 3′ → 5′ directionality (Fig. 4*D*) (45). Unwinding of Py_3-5_ resulted in an ∼6-fold increase in Cy3 emission. In contrast, Pu_3-5_ displayed only a 2-fold increase at identical unwinding conditions (Fig. 4*E* and *SI Appendix*, Fig. S7). From these values, the unwinding of Pu_3-5_ was estimated to be only 29% of that of Py_3-5_, and this was also confirmed by native PAGE (*SI Appendix*, Fig. S6). In vitro unwinding by PcrA under multiple turnover conditions is a slow process, and a timescale of ∼300 min at room temperature is comparable to that previously reported using a similar 10-fold excess of PcrA over substrate (48).

For the 3 enzymes, a calculation of the relative initial unwinding velocities for the 2 types of substrates revealed a similar trend to that observed for the unwinding efficiencies (Fig. 4*F*). Furthermore, XPD unwinding in the presence of 3 different trapping strands confirmed that DNA substrates carrying a homopurine sequence in the displaced strand are unwound with initial velocities that are between 60 to 40% lower than for homopyrimidine sequences (*SI Appendix*, Fig. S4*A* and Table S4). Finally, binding experiments also ruled out the possibility that the observed bias is induced by a differential affinity of the helicases for ss homopyrimidine or homopurine tracking strands (*SI Appendix*, Table S5).

The above observations are in agreement with the results reported by Taylor et al. (49) for the processive NPH-II RNA helicase. According to those results, a 3-fold increment of unwinding amplitude is observed when homopyrimidines are located on the 5′ displaced strand. Importantly, sequences with identical thermodynamic stability also show this behavior (49). Whereas Taylor et al.’s results could be regarded as an unusual set of observations, our analysis and experiments suggest that the molecular events underlying such a purine/pyrimidine bias can be straightforwardly related to the intrinsic dynamics of the duplex and are thus universal.

Taken together, the experimental characterization of the 3 model helicases confirms h-unwind predictions and demonstrates that the constructs with the homopyrimidine sequence at the displaced strand, namely Py_5-3_, Py_3-5_, were always more efficiently unwound, regardless the helicase type and directionality.

## Biological Implications

It is known that the sequence-dependent structure (50, 51) and stability (52) of DNA may contribute to modulate the activity of nucleic-acid processing machines (16–18, 39). However, asynchronous base-pair opening had never been tested in the context of helicase unwinding, and systematic studies were lacking on how the nucleic acid sequence could affect the base-pair intermediates during unzipping of ss/ds junctions. Our results linking asymmetric base-pair dynamics to unwinding efficiency support a mechanism where helicases are sensitive to the sequence composition in a way related not only to the stability of the base pair but also to the stability of the asymmetric intermediates involved during opening—thus further expanding the structural and functional complexity of the double helix. Specifically, helicases can more easily unwind substrates with pyrimidine-rich displaced strands, whether RNA (49) or DNA. Such purine/pyrimidine discrimination directly suggests that the sequence of nucleic acid duplexes contributes to regulating, as well as targeting, helicase activity in an orientation-dependent manner. That is, helicases can more easily unwind duplex portions if they proceed from one side than the other—hence bringing about an unanticipated level of gene regulation. For instance, the direction-dependent sequence bias may influence the efficiency with which sense and antisense strands of a genomic region are processed. Quantifying and knowing the most efficient unwinding direction of a specific duplex region should influence our thinking about how a specific helicase works and how it is used within the cell (*SI Appendix*, *Discussion*).

From an evolutionary perspective, the intrinsic dynamics of nucleic acids may represent a biasing factor on the evolution of helicase mechanisms (53, 54). For RNA, the relative stability of dangling intermediates shown in Fig. 1*D* conveys that the opening of base pairs in A-RNA naturally follows a specific directionality, namely passing through 5′ flipped-out intermediates. Therefore, one may argue that helicases evolved to unwind RNA would prevalently show one specific unwinding directionality. On the other hand, the opening of base pairs in B-DNA includes either 3′ or 5′ flipped-out intermediates (depending on the sequence), and one may argue that this bimodality may have left a signature in the evolution of DNA helicases. It is therefore intriguing to note that, among well-characterized RNA helicases, 3′ → 5′ directionality seems to have prevailed along evolution, as they are overrepresented when compared to DNA helicases, which, in contrast, show a more balanced ratio between 3′ → 5′ and 5′ → 3′ directionality (*SI Appendix*, Table S8). We speculate that the directionality selected for RNA and DNA helicases along evolution reflects the different intrinsic dynamics of RNA and DNA base pairs.

Overall, the sequence bias and direction-dependent efficiency of unwinding reported here may confer an additional layer of complexity for the evolutionary fine-tuning of genome function and cell-cycle regulation.

## Conclusions

By gaining access in a systematic manner to the atomistic spatiotemporal details of the unwinding process, we show that the opening of a base pair follows a stepwise mechanism whose directionality is modulated by the sequence and that, being controlled by the extension of stacking interactions, differs in A- and B-helices. Furthermore, we reveal that the general unzipping model stating the unwinding preference of helicases solely dependent on the thermodynamic stability of the substrate should be amended to include both the directionality of the helicase and the strand-specific nucleobase dynamics of the double helix. However, note that the simulations and experiments presented herein were performed on relatively short duplexes and that the model should be further extended in order to consider the probability of bubble formations in longer duplexes. A key feature of the unwinding model is that the population of stacked nucleobases at the displaced strand represents a bottleneck (or barrier) for helicase activity. As we have shown, the experimentally observed purine/pyrimidine bias finds a direct explanation in the different kinetics of base-pair opening rather than in the stability of the base pair itself. From a functional perspective, such a bias may represent yet another level in the fine-regulation of genome function. Our results offer motivation and a challenge for the design of future experimental research on the mechanisms of helicases and of duplex opening, and ultimately on the modulation of gene expression by the direction-dependent unwindability of the target sequence. We envision that the asymmetric unwinding model will enable characterization of the influence of the intrinsic dynamics of nucleic acids on the functioning of other helicase superfamilies and nucleic-acid processing machineries, thus furthering our understanding of gene regulation.

## Methods

### Computational Model and Unwinding Simulations.

We studied hexameric A-type RNA and B-type DNA canonical duplexes represented with an all-atoms structure-based Hamiltonian generated with SMOG (55). Multiple opening and closure events of RNA and DNA duplexes were achieved by applying a constant force, *f*_*C*_ = 14 pN, between the 3′ and 5′ hydroxyl groups of the terminal base pair (Fig. 1*A*) and running Langevin dynamics with GROMACS (56) and PLUMED (57). The bias in the population distribution due to the application of the external constant force *f*_*C*_ was removed by applying the reweighting procedure described in *SI Appendix*, *Methods*. Note that, although the exact mechanism of base-pair opening could depend on the choice of the biased variable, the reweighting procedure ensures that the computed relative stability of the 5′ and 3′-dangling intermediates (Fig. 1*D*) is not dependent on this choice. Further analysis of the pathway population sampled during the opening of base pairs is reported in *SI Appendix*, Fig. S1 and the related *SI Appendix*, *Supplementary Text*. Full methodological details are in *SI Appendix*. PLUMED input files are available on PLUMED-NEST (www.plumed-nest.org) as plumID:19.074.

### Protein Expression and Purification, DNA Labeling and Unwinding.

XPD from *Thermoplasma acidophilum*, PcrA from *B. stearothermophilus* (gift from Mark S. Dillingham, School of Biochemistry, University of Bristol, University Walk, Clifton, United Kingdom), and RecD2 from *Deinococcus radiodurans* (gift from Dale Wigley, Faculty of Medicine, Department of Infectious Disease, London, United Kingdom) were expressed and purified as described in *SI Appendix*, *Methods*. Labeled and unlabeled DNA strands (*SI Appendix*, Table S1) were purchased from IDT. Dry DNA pellets were resuspended in 50 mM tris(hydroxymethyl)aminomethane⋅HCl (pH 7.5) to a final concentration of 100 μM and stored at −20 °C. The DNA constructs were hybridized and purified as reported elsewhere (58) (*SI Appendix*, *Methods*). DNA-unwinding assays were performed using the kinetic scan option of a Cary Eclipse spectrophotometer (Agilent Technologies LDA UK Ltd.). Assays were carried out with excitation at 547 nm and emission collected at 565 nm. ATP concentration was 1, 5, and 0.1 mM for XPD, RecD2, and PcrA unwinding assays, respectively. Full experimental details are in *SI Appendix*, *Methods*. The research data underpinning this publication can be accessed at https://doi.org/10.17630/84c3a74e-eb89-4b37-a3ed-cd1cf0feeae3.

## Supplementary Material

Supplementary File
