# Supplementary material for: Asymmetric base-pair opening drives helicase unwinding dynamics
Source: Proc Natl Acad Sci U S A. 2019 Oct 18;116(45):22471–7. doi: 10.1073/pnas.1901086116 (PMC6842596; doi:10.1073/pnas.1901086116)
Supplement: Supplementary File [file pnas.1901086116.sapp.pdf]

PNAS
www.pnas.org

Supplementary Information for

Asymmetric base pair opening drives helicase unwinding dynamics

Francesco Colizzi[1,5*], Cibran Perez-Gonzalez[2], Remi Fritzen[2], Yaakov Levy[3], Malcolm F. White[2], J. Carlos Penedo[2,4*], and Giovanni Bussi[1*]

[1] SISSA – Scuola Internazionale Superiore di Studi Avanzati, Trieste, Italy;
[2] School of Biology, BSRC complex, University of St. Andrews, St. Andrews, UK;
[3] Department of Structural Biology, Weizmann Institute of Science, Rehovot, Israel;
[4] SUPA School of Physics and Astronomy, University of St. Andrews, St. Andrews, UK;
[5] Institute for Research in Biomedicine (IRB Barcelona), The Barcelona Institute of Science and Technology (BIST), Barcelona, Spain.
*Email: cecio.colizzi@gmail.com; jcp10@st-andrews.ac.uk; bussi@sissa.it

This PDF file includes:

Part I. Supplementary Data

Part II. Supplementary Methods

Part III. Supplementary Discussion

Figures S1 to S10

Tables S1 to S9

SI References

www.pnas.org/cgi/doi/10.1073/pnas.1901086116

Part I. Supplementary Data

Analysis of base pair opening pathways

The path population sampled during the opening of a base pair is reported in Figure S1. We characterized and quantified three possible scenarios: 1) the transition of the base pair from a closed state (C) to an open state (O) passing through a 5'-dangling intermediate; 2) the transition of the base pair from C to O passing through a 3'-dangling intermediate; 3) the direct transition of the base pair from C to O. The bias of the external constant force was not removed from the path populations shown. It is however remarkable the similarity between the kinetics data shown in Figure S1A and the thermodynamic data reported in Figure 1d of the main text. The relative population of conformers is not significantly influenced by the bias as the external force acted similarly on both 3'- and 5'- dangling intermediates as suggested by the similar average weight (w) of 3'- and 5'- dangling intermediates (Figure S1C).

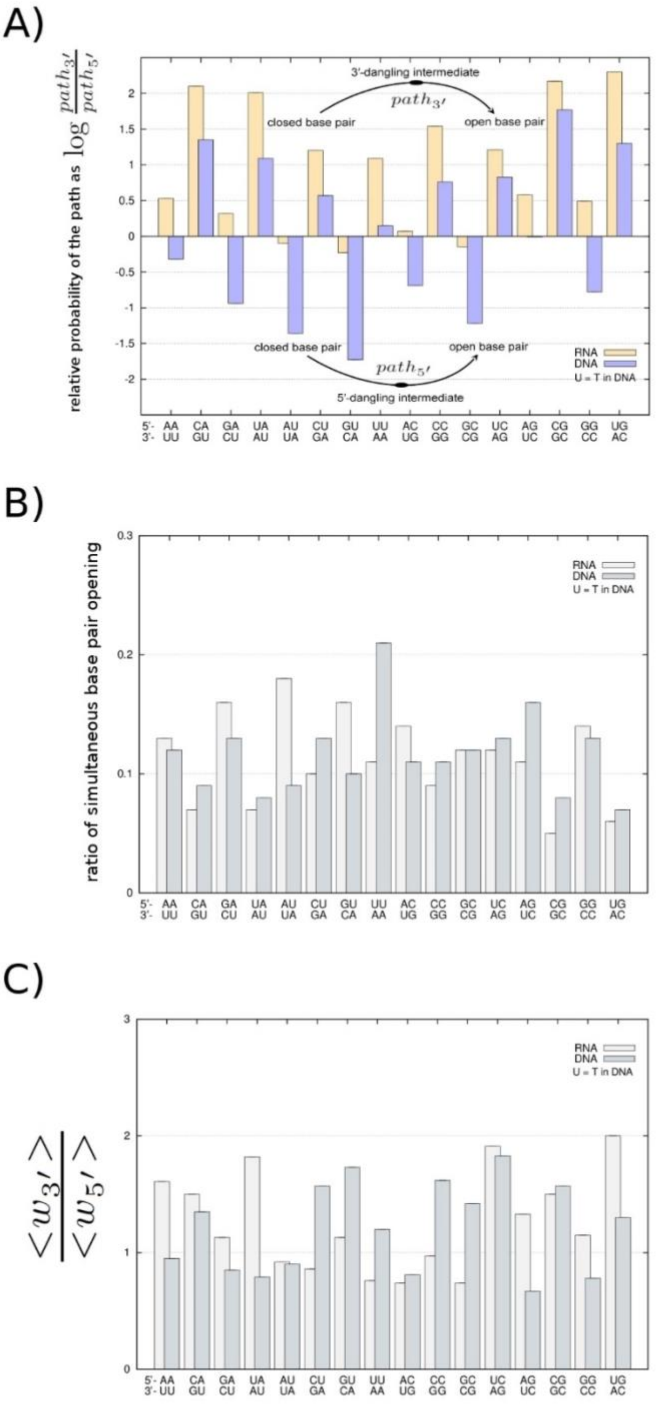

Figure S1. Comparison among base pair opening pathways. A) Relative probability of the 3'- and 5'-unwinding pathway sampled during the simulations. The plot shows the ratio between the actual occurrence of base pair opening (i.e. from Closed to Open state) processes passing through 5'- or 3'- dangling intermediates ($path_{5'}$ and $path_{3'}$ respectively). The plot shows the sampled populations during the opening of the left base pair in each nearest-neighbor combination. RNA base pair combinations largely populate the upper half of the plot showing a preference for 3'-dangling intermediates during RNA unwinding. In contrast, both 3'- and 5'- dangling intermediates are populated in the unwinding of DNA duplexes. B) The fraction of 3'- and 5'-simultaneous base pair opening is shown for each nearest-neighbor combination. C) Relation between the average weight of 3'- and 5'- dangling intermediates.

Labelled Oligonucleotides constructs

Table S1. Cy3 and Dabcyl (Dab) labelled oligonucleotides used to characterize the unwinding efficiency of 5’→3’ and 3’→5’ helicases by fluorescence quenching

Helicase polarity	Strand (Construct name)	Sequence (5’ to 3’)
**5’→3’**	*ssPu53_Dab (Py5-3)*	AGCTACCATGCCTGCACGAATGAGGAAGGGAGGAAAGGAAGA-Dab
	ssPy53_Cy3 (Py5-3)	Cy3-TCTTCCTTTCCTCCCTTCCTC
	ssPy53_Dab (Pu5-3)	AGCTACCATGCCTGCACGAATCTCCTTCCCTCCTTTCCTTCT-Dab
	ssPu53_Cy3 (Pu5-3)	Cy3-AGAAGGAAAGGAGGGAAGGAG
**3’→5’**	*ssPu35_Cy3 (Py3-5)*	Cy3-AGAAGGAAAGGAGGGAAGGAGTAAGCACGTCCGTACCATCGA
	ssPy35_Dab (Py3-5)	CTCCTTCCCTCCTTTCCTTCT-Dab
	ssPy35_Cy3 (Pu3-5)	Cy3-TCTTCCTTTCCTCCCTTCCTCTAAGCACGTCCGTACCATCGA
	ssPu35_Dab (Pu3-5)	GAGGAAGGGAGGAAAGGAAGA-Dab

Table S2. 6-carboxyfluorescein (FAM) labelled oligonucleotides used to characterize helicase binding to homopyrimidine, homopurine and mixed sequences using fluorescence quenching (XPD) and fluorescence anisotropy (PcrA and RecD2)

	Strand	Sequence (5’ to 3’)
	**FAM_Py**	FAM-TCTTCCTTTCCTCCCTTCCTC
	**FAM_Pu**	FAM-AGAAGGAAAGGAGGGAAGGAG
	**FAM_Mix**	FAM-AGCTACCATGCCTGCACGAAT

Table S3. Oligonucleotide trapping sequences used for XPD unwinding

Substrate	Pu_{5-3}	Py_{5-3}
**Method 1**	5’-GAGGAAGGGAGGAAAGGAAGA	5’-CTCCTTCCCTCCTTTCCTTCT
**Method 2**	5’-TTCCCTCCTTTC	5’-AAGGGAGGAAAG
**Method 3**	5’-CTCCTTCCCTCCTTTCCTTCT	5’-GAGGAAGGGAGGAAAGGAAGA

Table S4. Initial unwinding velocities obtained for XPD, PcrA and RecD2 and the specified DNA substrates

Helicase	DNA substrate	Initial velocity (min^{-1})
XPD (No trap)	Pu_{5-3}	2.14 ± 0.01
XPD (No trap)	Py_{5-3}	3.41 ± 0.03
XPD (Trap method 1)	Pu_{5-3}	0.82 ± 0.02
XPD (Trap method 1)	Py_{5-3}	1.41 ± 0.04
XPD (Trap method 2)	Pu_{5-3}	0.56 ± 0.05
XPD (Trap method 2)	Py_{5-3}	1.50 ± 0.03
XPD (Trap method 3)	Pu_{5-3}	0.43 ± 0.02
XPD (Trap method 3)	Py_{5-3}	1.18 ± 0.03
PcrA	Pu_{3-5}	0.005 ± 0.001
	Py_{3-5}	0.022 ± 0.004
RecD2	Pu_{5-3}	0.0042 ± 0.0002
	Py_{5-3}	0.0097 ± 0.0007

Table S5. Values of K_D and Hill coefficient obtained for the three helicases investigated by fitting the experimental binding curve to the Hill model equation $S = S_0 + \Delta S[x^n]/[k_D^n + x^n]$.

Helicase	ssDNA sequence	K_D	Hill coefficient (n)
**XPD**	FAM_Py	14 ± 1	2.2 ± 0.2
	FAM_Pu	278 ± 50	0.81 ± 0.05
	FAM_Mix	18 ± 2	1.9 ± 0.1
**PcrA**	FAM_Py	34 ± 4	0.85 ± 0.05
	FAM_Pu	160 ± 20	1.0 ± 0.2
	FAM_Mix	44 ± 6	0.86 ± 0.04
**RecD2**	FAM_Py	32 ± 1	1.08 ± 0.03
	FAM_Pu	445± 54	0.92 ± 0.06
	FAM_Mix	155 ± 2	1.0 ± 0.1

Strand-swapped DNA duplexes exhibit identical thermodynamic stability.

Here we describe the melting experiments performed to confirm that the presence of the fluorophore and the overhang did not alter the thermodynamic stability of the strand-

swapped constructs. In the experimental configuration used, duplex DNA melting is reported as an increase in Cy3 emission as the fluorophore distance to the Dab quencher increases due to strand separation (Figure S2a). The relative thermodynamic stability of the different dye-quencher constructs was determined at ionic strength conditions and buffer solutions identical to those used for the unwinding. The melting temperatures (T_m) obtained for both 5′ → 3′ substrates were very similar with values of 62.3 ± 0.3 ºC and 62 ± 0.2 ºC for Pu_{5-3} and Py_{5-3}, respectively (Figure S2b). The corresponding melting profiles of the 3′ → 5′ substrates, Pu_{3-5} and Py_{3-5}, also confirmed that the constructs had the same thermodynamic stability with T_m values of 67.1 ± 0.1 ºC for Pu_{3-5} and 67.6 ± 0.2 ºC for Py_{3-5} (Figure S2c). The relative increase in T_m values (ΔT_m ~ 5 ºC) observed for Pu_{3-5} and Py_{3-5} compared to Pu_{5-3} and Py_{5-3} agrees with previously reported stabilization effects induced by the different ionic background used (see Methods)[54].

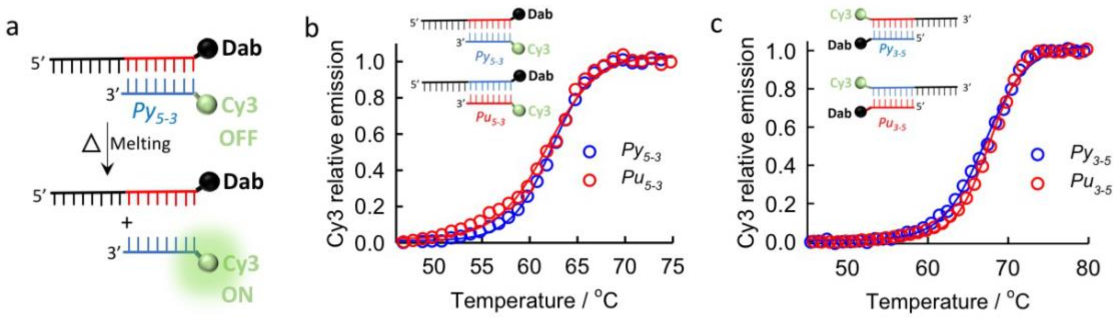

Figure S2 Thermodynamic stability of strand-swapped duplex DNA substrates. (a) Schematic of the fluorescence quenching assay used to obtain the temperature melting profiles of the two pairs of DNA constructs employed to investigate helicase unwinding. The duplex DNA regions are composed of 21 base pairs and the substrates are named according to the nucleobase composition of the displaced strand and the polarity of a 21-nt single-stranded DNA overhang. For instance, Pu_{5-3} denotes a duplex DNA substrate comprising a 21-nt 5′ overhang and a homopurine sequence on the displaced strand (see Supplementary Table S1 for details and Supplementary Methods section). The close proximity of Dab to the Cy3 fluorophore quenches its fluorescence emission, which is restored due to strand separation during melting. (b) Melting profiles obtained for Py_{5-3} (blue) and Pu_{5-3} (red) duplexes employed as substrates for helicase unwinding with 5′ → 3′ polarity. (c) Melting profiles obtained for Py_{3-5} (blue) and Pu_{3-5} (red) duplexes employed as substrates for helicase unwinding with 3′ → 5′ polarity. Solid lines represent the fitting of the experimental data to a Boltzmann sigmoidal equation from which T_m values were obtained.

Unwinding gel assays

XPD activity determined by activity gel assays was performed with DNA substrates carrying the Cy3 dye but lacking the Dabcyl quencher (Table S1). The reaction mix containing DNA and XPD helicase was incubated for 10 minutes. This solution was then

split in three different tubes. One tube was separated as a control and ATP was added to the other two samples with final concentrations of 100 μM and 1mM ATP for XPD. As a control for the migration of ssDNA strands labelled with Cy3, ssDNAs were diluted to a final concentration of 50nM in helicase buffer (20mM MES pH 6.5, 0.1 mg/mL bovine serum albumin (BSA) and 1mM MgCl2). This mix was incubated for 10 minutes and the reaction was started by addition of either 100 μM or 1mM ATP with a final volume of 150 μL.

The XPD unwinding assay was stopped at plateau conditions (25 min for 1mM ATP and 50 min for 100 μM ATP) by addition of 10 μL of the reaction mix to 10 μL of stop solution (10mM Tris-HCl pH 7.5, 5mM EDTA, 0.5% SDS, 1 mg/mL Proteinase K). The samples were then incubated for 15 min at room temperature. Furthermore, 10 μL of control mixtures were also added to 10 μL of stop mix with further incubation at RT for 15 minutes. Finally, 5 μL Ficoll 15% were added to 10 μL of the previous solutions for a final DNA concentration of 0.25 pmole.

The unwinding efficiency was investigated by gel electrophoresis in a nondenaturing 12% (w/v) polyacrylamide gel with 240 μL APS and 60 μL TEMED. Gels were pre-run for 1 h in Tris/Borate/EDTA buffer (TBE) at 110V before DNA was loaded. Gels were visualized in a Typhoon FLA 7000 (GE Healthcare Life Sciences, Little Chalfont, UK) gel imaging system with excitation at 532 nm. The ssDNA and dsDNA bands were associated by comparison with the migration profile of the controls. The intensity of each band was then extracted using the 1D tool software provided with manual background subtraction.

Unwinding of RecD2 (Figure S5) and PcrA (Figure S6) using gel-based assays was performed following a similar protocol as described for XPD but replacing the reaction buffer for the appropriate one for each helicase as described in the main text. For RecD2, we observed a significant degree of complex precipitation when adding SDS in the stop buffer that was absent when SDS was removed from the stopping mixture (Figure S5).

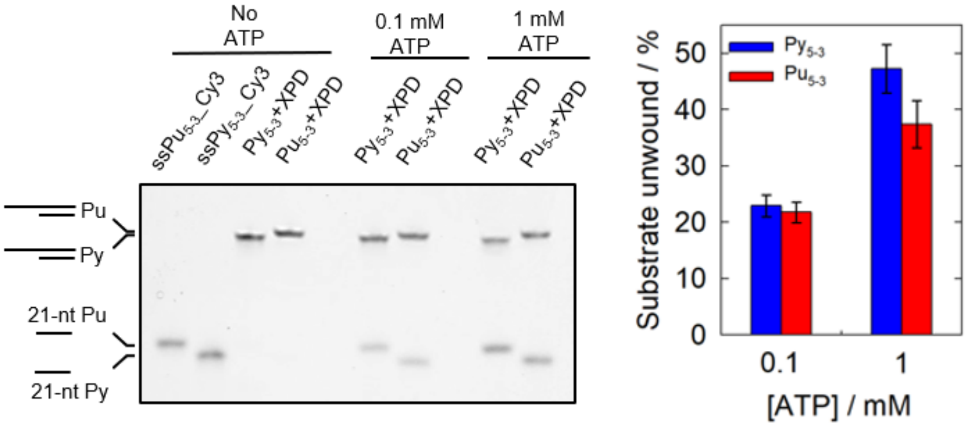

Figure S3. (Left) Representative activity gel assay to quantify the unwinding amplitudes of XPD $Py_{5\text{-}3}$ (blue) and XPD $Pu_{5\text{-}3}$ (red) duplexes at 100 µM and 1mM ATP. Reactions were stopped at plateau conditions (25 min for 1mM ATP and 50 min for 100 µM ATP) by addition of stop mix. (Right) Unwound substrate obtained at each ATP concentration. Data represent the mean for three replicates and error bars represent the SEM. DNA substrates are named according to the composition of the displaced strand (i.e. $Py_{5\text{-}3}$ indicates an only-pyrimidine displaced strand).

XPD unwinding in the presence of trapping strands

Helicase unwinding experiments in the presence of trapping strands were performed using the same protocols as described in the main text but including a 10-fold molar excess of the oligonucleotide sequence corresponding to each trapping method (see Table S4). Trapping strands were mixed with ATP beforehand and the reaction was started by addition of this mixture to a pre-incubated DNA-helicase complex. Experiments for each DNA substrate were repeated in triplicate and used to calculate the average relative unwinding amplitude, initial velocities and the corresponding standard errors.

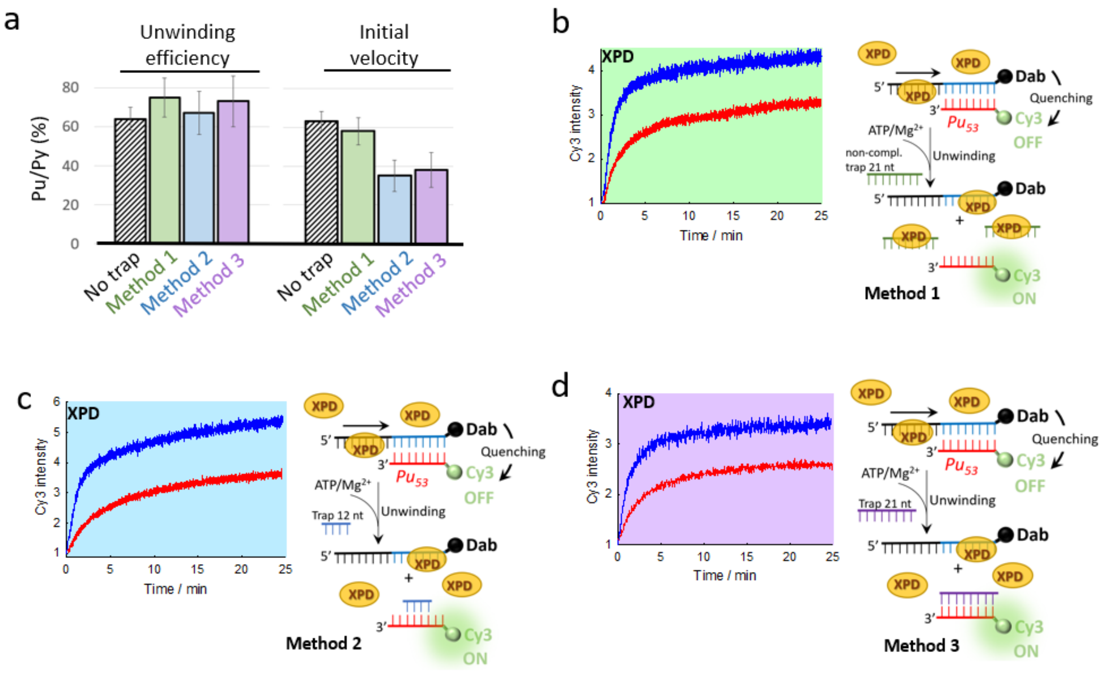

Figure S4. (a) Percentage XPD unwinding amplitude and initial velocity observed for homopurine sequences in the displaced strand relative to homopyrimidine sequences using no trapping strand (pattern) and three different trapping methods. Method 1 (green): the addition of 10-fold excess of a non-complementary and unstructured 21 nt oligonucleotide. Method 2 (blue): addition of a 12 nt oligonucleotide that partially complements the displaced strand. Method 3 (purple): addition of a 21 nt oligonucleotide that fully complements the displaced strand. (b-d) Normalized variation in fluorescence intensity of Cy3 as a function of time induced by the addition of 5 mM ATP and trapping

strand to pre-formed $Py_{5\text{-}3}$-RecD2 (blue) and $Pu_{5\text{-}3}$-RecD2 (red). Schematics of the trapping methods are shown next to the corresponding unwinding profile. Error bars represents the standard error of the mean of the three replicates for each experimental condition.

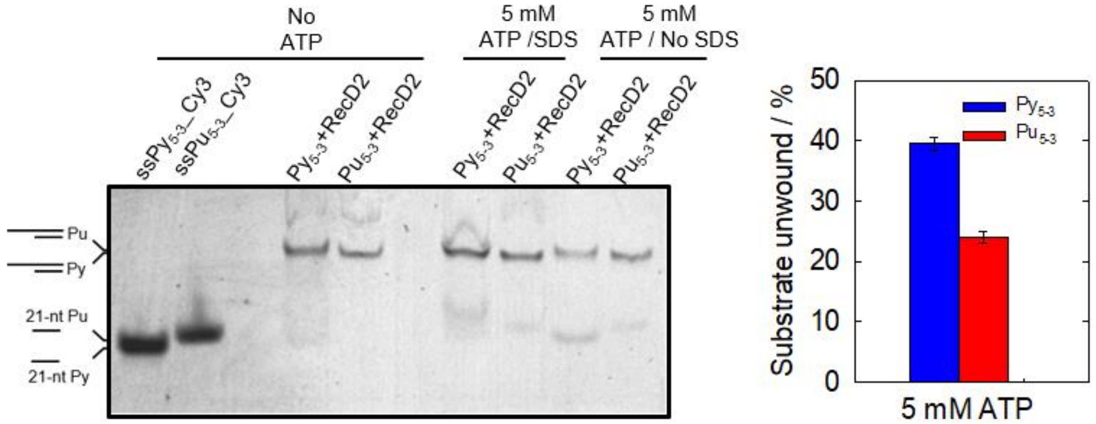

Figure S5. (Left) Representative activity gel assay to quantify the unwinding amplitudes of RecD2 $Py_{5\text{-}3}$ (blue) and RecD2 $Pu_{5\text{-}3}$ (red) duplexes at 5mM ATP. Reactions were stopped at plateau conditions (60 min) by addition of stop mix (see supplementary methods). (Right) Unwound substrate obtained 5 mM ATP for each DNA substrate. Data represent the mean for three replicates and error bars represent the SEM. DNA substrates are named according to the composition of the displaced strand (i.e. $Py_{5\text{-}3}$ indicates an only-pyrimidine displaced strand).

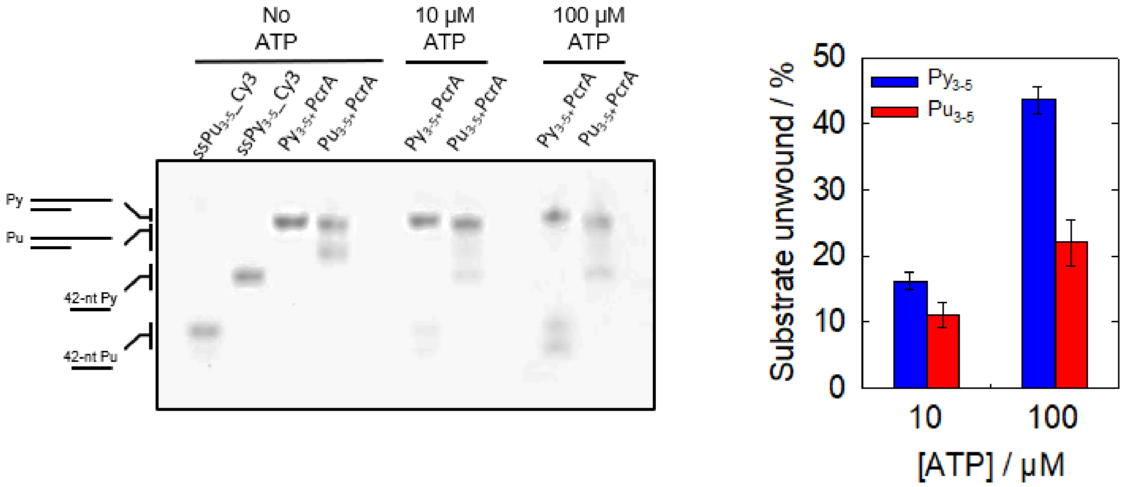

Figure S6. (Left) Representative activity gel assay to quantify the unwinding amplitudes of PcrA $Py_{3\text{-}5}$(blue) and PcrA $Pu_{3\text{-}5}$ (red) duplexes at 10 and 100 µM ATP. Reactions were stopped at plateau conditions (500 min for 10 µM ATP and 300 min for 100 µM ATP) by addition of stop mix (See Supplementary Methods). (Right) Unwound substrate obtained at each ATP concentration. Data represent the mean for three replicates and error bars represent the SEM. DNA substrates are named according to the composition of the displaced strand (i.e. $Py_{3\text{-}5}$ indicates an only-pyrimidine displaced strand).

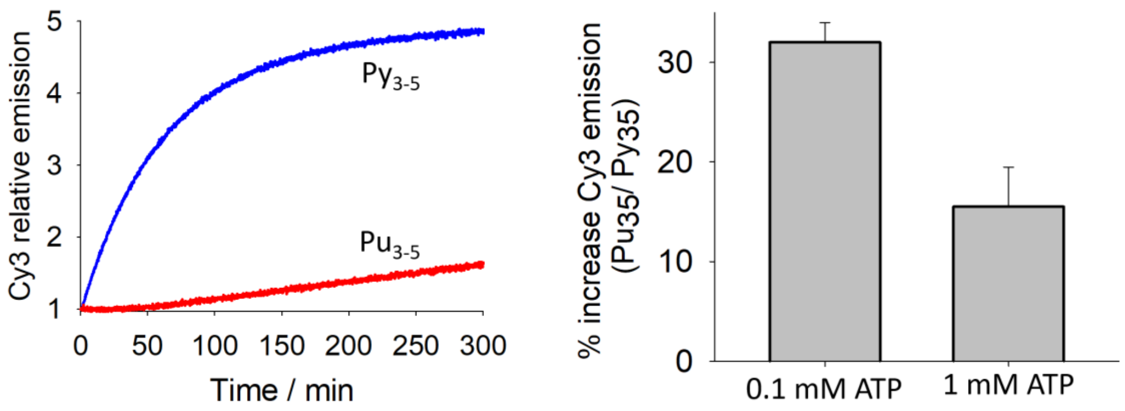

Figure S7. (Left) Normalized variation in Cy3 emission as a function of time following the addition of 500 nM PcrA to Py_{3-5} (red) and Pu_{3-5} (blue) duplexes. The concentration of ATP and Mg^{2+} was 0.1 mM and 1 mM, respectively. (Right) Relative increase in Cy3 emission between Pu_{3-5} and Py_{3-5} substrates induced by the addition of 500 nM PcrA to initiate unwinding at the indicated concentrations of ATP. DNA substrates are named according to the composition of the displaced strand (i.e. Py_{3-5} indicates an only-pyrimidine displaced strand).

DNA binding assays (see Supplementary Discussion)

HPLC purified oligonucleotides carrying a 5' 6-(carboxyfluorescein) modification (FAM) were purchased from Integrated DNA Technologies (IDT, USA) and their sequences are shown in Table S2. XPD binding isotherm was obtained using fluorescence quenching between the iron sulfo-cluster domain and the fluorophore as previously reported[1]. For PcrA and RecD2, a fluorescence anisotropy assay was carried out using a Varian Cary Eclipse equipped with automated polarizers as previously described[2]. All experiments were carried out in identical buffers as those used in the helicase assays but with no ATP. Oligonucleotide concentration was assessed by absorption spectrometry. FAM-labelled substrates were used at 10 nM concentration for quenching and anisotropy assays. The protein concentration was increased cumulatively with corrections made for dilution. Anisotropy experiments were repeated three times with excitation at 494 nm and emission at 520 nm corresponding to excitation and emission maxima of the FAM dye. Binding isotherms were fitted to a Hill model using the following expression as previously described[3] using the following expression: $S = S_0 + \Delta S[x^n]/[k_D^n + x^n]$, where S represents either percentage quenching or anisotropy, and S_0 and ΔS indicate the signal in the absence of protein and the total change at saturating protein concentration, respectively. K_D represents the dissociation constant in nM and n is the Hill coefficient. The program used to fit the data was Sigma Plot v.14. The values obtained for the K_D and n are listed in Table S5. The values of the Hill coefficient (n) obtained for RecD2 and PcrA are close to unity for all sequences investigated. For XPD, we obtained values of

n~2. This agrees with previously reported values ranging from n~1.8 for wild-type XPD to n~4 for certain XPD variants[3].

Part II. Supplementary Methods

Nucleic acids model

Structure-based and minimal models simplify the underlying free energy of a biomolecular system thus allowing the sampling of significant conformational states and capturing the essence of the process of interest[4–6]. Structure-based models have been widely used to rationalize and predict experimental folding trends and mechanisms in proteins and nucleic acids[5–7]. The use of a coarse-grained structure-based model is not merely a convenience in terms of simulations time. In this work a model based on the geometry of the native structure was used so as to emphasize the fundamental difference between A-type RNA and B-type DNA helices and capture the structural determinants of the (un)winding mechanism. Recently, we have shown that a minimalist representation of RNA structures including relative base-base arrangement alone can capture the main interactions that are relevant for describing RNA structure and dynamics[8].

We studied the formation and rupture of hexameric A-type RNA and B-type DNA duplexes with canonical Watson and Crick base pairs. Six A-type RNA and six B-type DNA duplexes were built using the *make-na* server[78]. The sequence (Table S7) of each duplex was designed so as to allow the 16 nearest-neighbor base-pair combinations to be investigated employing different pulling schemes.

Here, we used a structure-based (Gō) model in which attractive Lennard-Jones interactions are used to represent native contacts, whereas all other interactions are repulsive terms[9]. All heavy atoms were explicitly represented and the corresponding structure-based potentials for the DNA and RNA duplexes were generated using the SMOG code 1.1.1 [9–11]. All but the cutoff default values were used. Inter-strand base interactions are known to play an important role in nucleic acids duplexes dynamics and, to include those interactions into our simulations, native contacts were assigned to all atom-atom pairs that are within 5 Å in the native structure. In this set up, the ratio between the number of adjacent stacking and hydrogen bonding contacts is equivalent to the one at the default cutoff value of 4 Å.

Unwinding simulations and analysis

In the spirit of laser optical tweezer experiments, we investigated the opening and closure of RNA and DNA duplexes by applying a constant force, f_C= 14 pN, between the 3′ and 5′ hydroxyl group of the terminal base pair of the duplex (Figure 1a). The temperature of

each system was modulated so that the nucleic acid could spontaneously hop between folded and unfolded configurations with similar probability (Figure 1b, Table S7). In optical-tweezer experiments performed at 300 K and at a constant force of about 14 pN a similar probability of folded and unfolded states can be observed for small nucleic acid hairpins[83,84]. Langevin dynamics with constant force acting on the duplex termini was performed with GROMACS 4.0.7[85] combined with PLUMED 1.3[86]. The free-energy differences shown in Figure 1d were estimated from 2.56x10^9 steps of constant-force simulations with coordinates written every 1000 steps.

During the folding and unfolding of the duplex, basepair-opening intermediates dangling at the 3′ or 5′ strand of the ss/ds junction were defined by the combination of two collective variables (CVs): one accounting for the base pairing and the other for the base stacking (for dangling intermediate definition see "Definition of 3' or 5' dangling intermediates" below and Table S6). The bias in the population distribution due to the application of the external constant force f_C is removed by assigning to the configuration at time t the weight $w(t) = e^{-\beta d(t) f_c}$, where $d(t)$ is the end-to-end distance between the 3′ and 5′ terminal hydroxyl groups at time t, and $\beta = 1/k_B T$ is the inverse thermal energy. The removal of the bias is required since the stability of the relevant intermediates and, thus, the unwinding mechanism could be force dependent. The resulting unbiased free-energy profile can be reconstructed for any *a posteriori* chosen CV as $F(s) = -k_B T \ln \int dt \sum w(t) \delta(s - s(q(t))))$, where q are the microscopic coordinates and $s(q)$ is the CV value for those coordinates. Here, we characterized the mechanism of unwinding for each nearest-neighbor combination of base pair by direct estimation of the free-energy difference between 5′- and 3′- dangling intermediates, $\Delta F = -k_B T \ln \int dt \sum_{t \in d5} w(t) / \int dt \sum_{t \in d3} w(t)$, where $d5$ and $d3$ are the set of snapshots satisfying the geometrical requirements for 5′ and 3′-dangling intermediates, respectively. Note that, although the exact mechanism could depend on the choice of the biased variable, the reweighting procedure ensures that the computed relative stability of the 5' and 3'-dangling intermediates is not dependent on this choice. Further analysis of the pathway population sampled during the opening of base pairs is reported in Figure S1 and the related Supplementary text.

Helix unwindability index (h-unwind)

We define the direction-dependent *helix unwindability* index as $1/\langle e^{-F/k_B T}\rangle$, where $k_B T$ is the thermal energy, F is the free-energy of stacked nucleobases at 3′ (*h-unwind*$_{5' \rightarrow 3'}$) or 5′ (*h-unwind*$_{3' \rightarrow 5'}$), and $\langle\ \rangle$ denotes the average over the sequence of base steps. Within this definition, we note that *h-unwind* is inversely related to the population of stacked nucleobases at the displaced strand—thus, the higher the *h-unwind* value, the higher the helicase unwinding efficiency.

Definition of 3’ or 5’ dangling intermediates.

For each nucleobase, the intracyclic nitrogen atom involved in the Watson and Crick base pair was used as reference to account for base pairing and base stacking (Figure S7A). The contacts between the nitrogen atom of two nucleobases were calculated using the coordination number in PLUMED. In particular, the threshold distance for defining the effective contact between two nitrogen atoms was evaluated from the maximum distance populated in the pair distribution function (Figure S7B) calculated from a 20M steps long unbiased trajectory obtained at the same reduced-unit Temperature (T) used during the unwinding simulations. Threshold distances are schematized in Table S6. The time step used in GROMACS was the default one for structure-based models (dt=0.0005 ps)[9–11]; note that the structure-based model is run in reduced units and the mass of carbon = 1.

In this metric, a 3’ dangling intermediate is counted if ST3 and HB2 contacts are formed and ST5 and HB1 are disrupted. Similarly, a 5’ dangling intermediate is counted if ST5 and HB2 contacts are formed and ST3 and HB1 are disrupted.

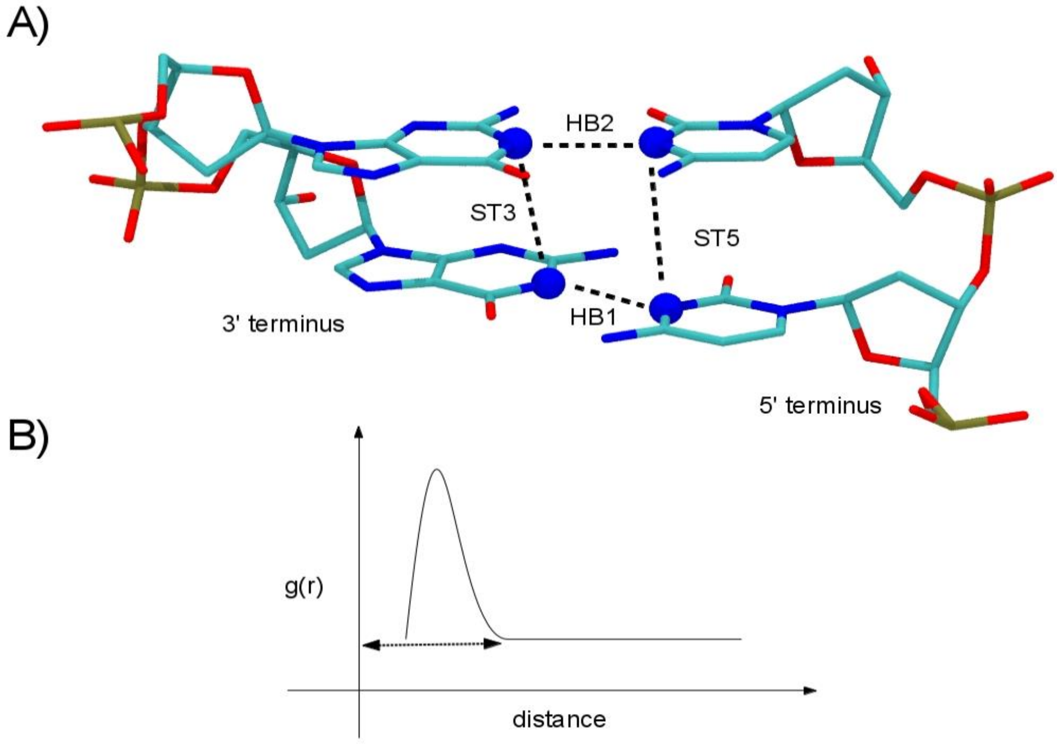

Figure S8. Definition of the collective variables used to describe base stacking and base pairing in RNA and DNA duplexes. A) Structural representation of the pairing (HB1, HB2) and stacking (ST3, ST5) contacts between intracyclic nitrogen atoms (blue spheres). Carbon atoms are in cyan, oxygen in red, nitrogen in blue, and phosphorus atoms in orange. B) Example pair distribution function used to define the pairing and stacking threshold distances for each nearest-neighbor contact.

ST3=ST5 (A-RNA/B-DNA)[a)]		0.75/0.7	0.8/0.75	0.75/0.7	0.9/0.8
	5′-	RR	RY	YY	YR
	3′-	YY	YR	RR	RY

Table S6. Threshold distances (nm) used for the nearest-neighbor contacts in A-RNA and B-DNA constructs. HB1=HB2 = 0.55 nm, both in DNA and RNA. [a)]In B-DNA, the threshold distance for the stacking between purines (RR) was ST_{RR}= 0.67 nm.

Sequence and parameters used during the unwinding of the DNA and RNA duplexes

We studied the formation and rupture of hexameric A-type RNA and B-type DNA helices with canonical Watson and Crick base pairs. Six A-type RNA and six B-type DNA duplexes were built using the *make-na* server[12]. The sequence of each duplex was designed so as to allow the 16 nearest-neighbor base-pair combinations to be investigated employing different pulling schemes (Table S7). Note that the structure-based model is run in reduced units; in Gromacs, kB = 0.00831451, and not 1. Thus, to have a reduced temperature of 1, one must use a Gromacs temperature of 1/0.00831451 = 120.2717 K. Within this metrics, the folding temperature of proteins, for instance, is usually between 100-120 K [4–6].

A-form RNA						
5′-GGGGGG-3′<br>3′-CCCCCC-5′	5′-CGCGCG-3′<br>3′-GCGCGC-5′	5′-AAAAAA-3′<br>3′-UUUUUU-5′	5′-UAUAUA-3′<br>3′-AUAUAU-5′	5′-GAGAGA-3′<br>3′-CUCUCU-5′	5′-UGUGUG-3′<br>3′-ACACAC-5′	
T=95	T=97	T=89	T=93	T=91	T=95	
B-form DNA						
5′-GGGGGG-3′<br>3′-CCCCCC-5′	5′-CGCGCG-3′<br>3′-GCGCGC-5′	5′-AAAAAA-3′<br>3′-TTTTTT-5′	5′-TATATA-3′<br>3′-ATATAT-5′	5′-GAGAGA-3′<br>3′-CTCTCT-5′	5′-TGTGTG-3′<br>3′-ACACAC-5′	
T=92	T=93	T=88	T=89	T=90	T=92	

Table S7. Sequences of the A-RNA and B-DNA constructs used for the unwinding simulations performed at the GROMACS Temperature T (in reduced units).

DNA hybridization and purification

The dsDNA substrates were annealed in Tris-HCl buffer containing a background of 50mM NaCl. Samples were heated in water bath to 80 ºC for 10 minutes followed by slow cooling overnight to room temperature. In order to separate any remaining ssDNA, the sample was purified by gel electrophoresis on a nondenaturing 12% (w/v) polyacrylamide gel with 240 µL ammonium persulfate (APS) and 60 µL tetramethylethylenediamine (TEMED). Gel purified DNA constructs were extracted from the gel using the crash and soak method and precipitated by addition of 10% (v/v) of 3M sodium acetate (pH 5.2) and 250% (v/v) of cold absolute ethanol, gently mixed and incubated overnight at -20 ºC. This mix was then centrifuged at maximum speed for 30 min in cold room. The supernatant was removed, and the DNA pellet was re-suspended in either 20 mM MES pH 6.5, for XPD helicase assays or 20 mM Tris-HCl pH 7.5 for PcrA and RecD2 experiments. It should be noted that in PcrA duplex substrates, the position of the quencher was swapped to the displaced strand because phosphoroamidite chemistries are only commercially available for Dabcyl incorporation group at the 3′ end of the oligonucleotide sequence.

Protein expression and purification.

XPD from Thermoplasma acidophilum (TacXPD), was expressed and purified as described previously[49,87] with the exception that all purification buffers were degassed by Argon to remove oxygen and protect the iron-sulfur cluster from oxidation. The recombinant plasmid pET22b with ampicillin resistance containing the PcrA sequence from Bacillus stearothermophilus was a gift from Mark S. Dillingham. PrcA was transformed in Escherichia coli (E. coli) BL21 (DE3) cells. The cells were grown in Luria-Bertani medium with 100 µg/ ml ampicillin at 37ºC. PcrA purification was performed as described elsewhere[88]. Recombinant RecD2 from Deinococcus radiodurans was a gift from Dale Wigley.

DNA melting experiments

DNA melting experiments were carried out using a heating rate of 0.2 ºC/min with data collection interval of 0.2 ºC and an integration time of 1 s for each data point. Thermal denaturation of 50nM Pu53 and Py53 duplexes was performed in 20 mM MES (pH 6.5) containing 1 mM MgCl2, whilst Pu35 and Py35 duplexes were characterized in 20mM Tris-HCl buffer (pH 7.4) containing 200mM NaCl and 1 mM MgCl2. A layer of mineral oil was deposited on top to prevent evaporation. Melting curves represent the mean of three

replicates with baseline correction of the double-strand DNA and single-strand DNA regions as previously reported[53,87]. The melting temperature was obtained by fitting the corrected experimental curve to a Boltzmann sigmoidal equation as described elsewhere[87].

DNA unwinding assays.

All DNA unwinding experiments were carried out at 20 ºC with an average integration time per data point of 0.5 s unless stated otherwise. Unwinding efficiency of XPD was measured in 20 mM MES, pH 6.5, 0.1 mg/ml bovine serum albumin (BSA) and 1 mM MgCl2, using 50 nM duplex and 500 nM XPD, as previously reported. RecD2 and PcrA unwinding kinetics was measured in 20 mM Tris-HCl buffer, pH 7.4, 0.1 mg/ml, 200 mM NaCl and 5 mM MgCl2 (RecD2) or 1 mM MgCl2 (PcrA) using 500 nM helicase concentration and 50 nM duplex. Each mix was incubated for 10 minutes and the reaction was started by addition of ATP unless stated otherwise. The unwinding rate was calculated as the initial reaction velocity from the slope of the linear part of the unwinding profile. The Sigma Plot v.14 software package was used to determine the slope.

Part III. Supplementary Discussion

Comparison between structure-based MD simulations and i) ultrafast spectroscopy experiments and ii) the analysis of structural databases

The extensive molecular simulations presented herein depict the opening of RNA and DNA base pairs as an asymmetric (directional) stepwise process. In RNA, we found the process driven by the unpairing of the base at the 5′ terminus of a ss/ds junction followed by the unstacking of the resulting 3′-dangling base. This finding is consistent with ultrafast spectroscopy experiments that have detected a larger subpopulation of stacked conformers for a 3′-dangling fluorescent purine probe when compared to the equivalent 5′-dangling one[21]. In line with these results, 3′-dangling bases have also been counted as the most abundant pattern observed at ss/ds junctions in large ribosomal RNA crystal structures[22].

Comparison between structure-based MD simulations and melting experiments

The free-energy differences reported in Figure 1d of the main text could be compared with optical melting experiments performed on RNA[23] and DNA[24] dangling ends. Optical melting experiments have shown that single nucleotides overhanging at the termini of a nucleic acid helix can increase the thermal stability of the duplex in a sequence- and orientation-dependent manner. A link between the stabilization of the duplex and the population of dangling intermediates described in this work might reside in the capability of the dangling nucleobase to stack over the closing base pair protecting its Watson and Crick hydrogen bonds from water exchange[25]. In this framework, those dangling nucleobases that highly populate conformations that stack over the adjacent hydrogen bonds would provide a larger contribution to duplex stabilization. Vice versa, those dangling nucleobases that populate conformations stacked over the adjacent closing base pair without overlapping with its Watson and Crick hydrogen bonds would provide little or no contribution to duplex stabilization. Whereas in B-DNA both 3’- and 5’-dangling ends can overlap with the neighboring hydrogen bonds, the 5′-dangling ends in A-RNA duplexes are projected away from those hydrogen bonds (see Figure 1f of the main text for structural references) and the population of stacked bases is likely poorly described by the little or null stabilization of RNA duplexes detected by melting experiments[21,23,25].

Here the free-energy difference between 5'- and 3'- dangling intermediates shown in Figure 1d of the main text is compared to the free-energy difference derived from optical melting experiments between 5'- and 3'- dangling ends (Figure S9). As an example for DNA, the free-energy difference shown in Figure 1d for the nearest-neighbor combination $\begin{matrix} 5'-\mathrm{GG}-3' \\ 3'-\mathrm{CC}-5' \end{matrix}$ is compared to the difference between the free-energy contribution of the 5'-dangling end $\begin{matrix} 5'-\mathrm{GG}-3' \\ 3'--\mathrm{C}-5' \end{matrix}$ and the one of the 3'-dangling end $\begin{matrix} 5'--\mathrm{G}-3' \\ 3'-\mathrm{CC}-5' \end{matrix}$ reported by Bommarito et al[24].

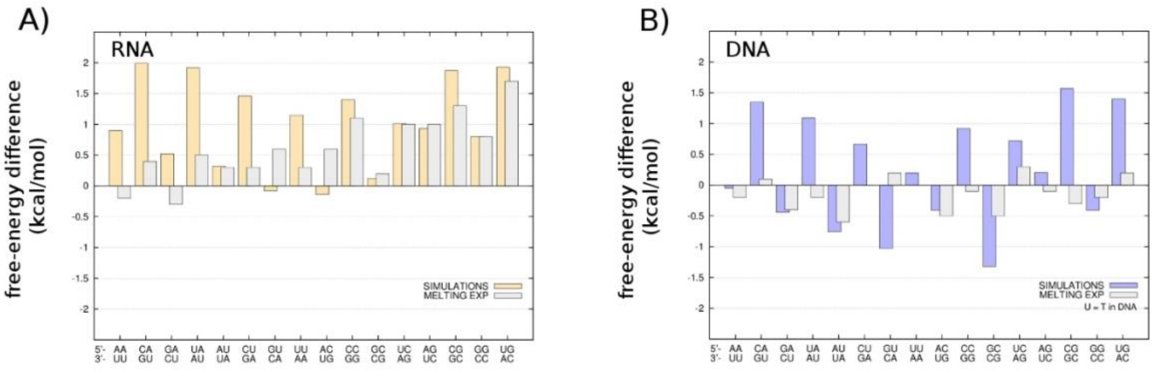

Figure S9. Free-energy difference between 5'- and 3'- dangling intermediates shown in Figure 1d of the main text against the free-energy difference derived from optical melting experiments between 5'- and 3'- dangling ends for the equivalent nearest-neighbor combination in (A) RNA[23] and (B) DNA[24].

Family	processing DNA		processing RNA		Class
	$5' \rightarrow 3'$	$3' \rightarrow 5'$	$5' \rightarrow 3'$	$3' \rightarrow 5'$	
DEAH/RHA		x		x	SF2
NS3/NPH-II		x		x	SF2
XPD/RAD3	x				SF2
Ski2-like		x		x	SF2
Suv3[(a)]				x	SF2
RIG1-like		x		x	SF2
RecQ-like		x			SF2
RecG-like		x			SF2
PcrA/UvrD/Rep		x			SF1
Pif1-like (Dda, RecD)	x				SF1
Upf1-like	x		x		SF1

[(a)]Despite the high level of conservation in eukaryotes, Suv3 helicase does not fall into any family.

Table S8 Processive superfamily (SF) 1 and 2 helicases with well-characterized polarity of translocation reported for RNA and DNA. The family classification follows the one of Fairman-Williams et al[29]. Table elaboration based on reffs[29–36]. Upf1-like helicases represent an interesting exception that is further discussed below.

Further discussion on the biological implication of asymmetric base pair dynamics

Here we report few examples where unusual homopurine/homopyrimidine sequences have been observed. Considering the essential role played by helicases in nearly all aspects of nucleic acid metabolism, the sequence bias and direction-dependent efficiency of unwinding reported in the manuscript may confer an additional layer of complexity for the evolutionary fine-tuning of genome function and cell-cycle regulation. Interestingly, homopurine/homopyrimidine sequences are present in eukaryotic genomes at higher than expected frequency[62]. Pu/Py motifs have been shown to modulate DNA replication[63] in an orientation-dependent manner[64], and participate in transcription regulation[65].

Upf1-family of RNA helicases translocating with 5′ → 3′ direction

An important exception to the trend in the RNA unwinding directionality highlighted in this work is the Upf1-like family of helicases that processes RNA duplexes in the unusual 5′ → 3′ direction and in a cofactor-dependent manner (Table S8). In the Upf1 protein, which plays a central role in mRNA surveillance processes, the interaction of an accessory domain with the cofactor Upf2 triggers conformational changes that allow efficient unwinding of target RNA duplexes[34,37]. Interestingly, Upf1 binds ssRNA in an orientation similar to that seen for prototypical 3′ → 5′ helicases[34], and the binding of Upf2 enables the unwinding process to run backward. In light of the energetics of duplex unwinding discussed in this study, the interaction between Upf1 and Upf2 could be interpreted as an evolutionary solution to enable 5′ → 3′ RNA processing and to overcome the otherwise energetically-discouraged displacement of nucleobases at the 3′ terminus in RNA ss/ds junctions. Notably, the need of a cofactor protein to enhance the efficiency of SF1 helicases that process RNA in a 5′ → 3′ direction is a common requirement also among the polyprotein enzymes with helicase activity discovered in Coronaviruses and Hepeviruses[38,39].

Finally, it is worth to mention that defining the effective polarity of helicase translocation is not trivial and several ambiguous examples have been reported in the literature. For instance, a 5′ → 3′ polarity had been initially assigned to the human SUV3[40]. However, recent experiments have clarified that SUV3 prefers substrates with a 3′ overhang over substrates containing a 5′ overhang or a blunt end and optimally unwinds RNA in the usual 3′ → 5′ direction[41], in agreement with our model. In this scenario, we speculate that the dominant 3′ → 5′ directionality observed in RNA-processing helicases and the dual, 3′ → 5′ and 5′ → 3′, directionality reported for the processing of DNA could be related to differences in the stepwise mechanism of helix unwinding disclosed in this study for RNA and DNA. The differential mechanism has in turn a direct explanation in the geometrical differences between A- and B-type helices.

Binding affinity between single stranded DNA and helicase (see Supplementary Methods).

Although the purine and pyrimidine constructs used in this study contain an identical 21 nt overhang to which the helicases bind at the ss-ds junction, we hypothesized that the observed differences in helicase efficiency could arise from variations in binding affinity to the homopyrimidine and homopurine sequences found in the translocating (tracking) strand during unwinding. To test this, we carried out binding assays using similar

conditions as those used in the unwinding experiments but in the absence of ATP. We compared the binding curves of each helicase to single-strand homopurine and homopyrimidine sequences, labelled at the 5' with carboxyfluorescein (FAM), matching the 21 nt translocating sequence used for duplex unwinding. A mixed sequence containing a 48% of purine bases (A and G) was also used for comparison. The presence of an iron-sulfur cluster domain in XPD has been shown to enhance the quenching of fluorescence of dyes positioned in the DNA[1] and we used this effect to monitor XPD affinity towards the three single strand DNA substrates (Figure S10a). For RecD2 and PcrA, we employed a fluorescence anisotropy assay that monitors relative changes in rotational diffusion of the dye attached to the DNA strand induced by protein binding (Figure S10b, and c panel). Our results clearly indicate that the three helicases studied are very sensitive to the nature of the oligonucleotide sequence (Figure S10d, see Supplementary Methods for fitting details). For XPD we observed a 20-fold increase in dissociation constant from ~14 ± 1 nM for the homopyrimidine sequence to a value of ~278 ± 50 nM for the homopurine analog. PcrA and RecD2 showed a similar affinity trend, with dissociation constant values for the homopyrimidine sequence of 34 ± 4 nM (PcrA) and 32 ± 1 nM (RecD2) that increased for the homopurine strand to values of 160 ± 20 nM and 445 ± 54 nM for PcrA and RecD2, respectively (Figure S10d). Dissociation constants for the mixed-sequence strand were similar to those obtained for the homopyrimidine sequence for XPD (18 ± 2 nM) and PcrA (44 ± 6 nM) and ~5-fold higher for RecD2 (155 ± 2 nM). Our data agree with previous studies where it has been shown that the binding constant for homopurine sequences of RecA[42], PriA[43] and UPF1[44] helicases is more than order of magnitude lower than for identical length pyrimidine sequences. It is important to note that the observed influence of base composition on helicase unwinding efficiency and binding affinities follows reverse trends. Whereas homopurine sequences on the translocating strand favor helicase unwinding activity, they exhibit lower binding affinities compared to homopyrimidine sequences. A similar trend has been observed for translocation by the Hepatitis C virus NS3h helicase[45], and more recently, for the *E. coli* UvrD helicase[46], but no such correlation has been reported in the context of helicase unwinding.

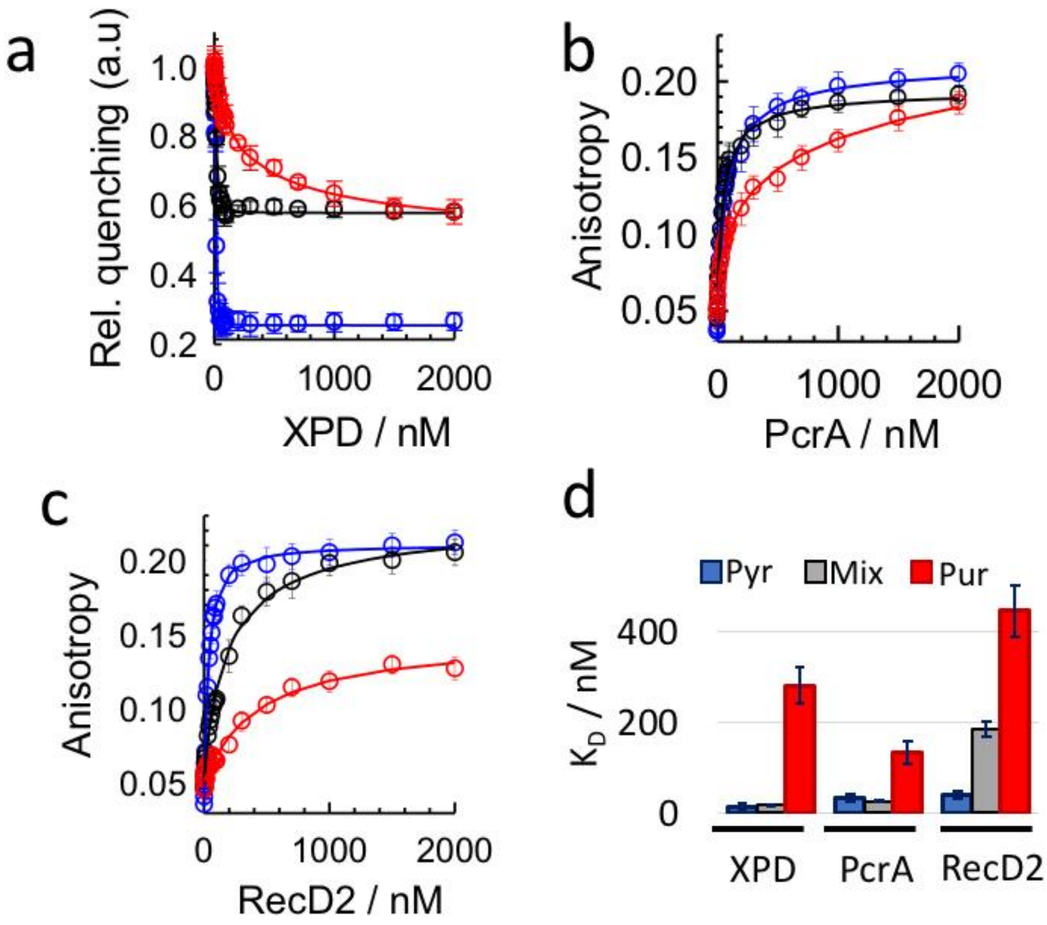

Figure S10 Binding affinity between single stranded DNA and helicases. (a) Binding isotherms of XPD, PcrA (b) and RecD2 (c) helicases to 5' FAM labelled homopyrimidine (blue), homopurine (red) and mixed (grey) single-stranded DNA oligonucleotides. Homopurine and homopyrimidine sequences matched the 21 nt sequence of the translocating strand used in the duplex unwinding assays. XPD binding was monitored by fluorescence quenching and for PcrA and RecD2 by anisotropy. Experimental conditions were identical to those used for unwinding but with no ATP added. Solid lines represent the fitting to a Hill model (see Supplementary section). (d) Comparison of dissociation constants obtained for the three helicases as a function of strand sequence. Error bars represent the standard deviation of three replicates.

SI References

1. Constantinescu-Aruxandei, D., Petrovic-Stojanovska, B., Penedo, J. C., White, M. F. & Naismith, J. H. Mechanism of DNA loading by the DNA repair helicase XPD. *Nucleic Acids Res.* **44,** 2806–2815 (2016).

2. Northall, S. J. *et al.* DNA binding and unwinding by Hel308 helicase requires dual functions of a winged helix domain. *DNA Repair (Amst).* (2017). doi:10.1016/j.dnarep.2017.07.005

3. Fan, L. *et al.* XPD Helicase Structures and Activities: Insights into the Cancer and Aging Phenotypes from XPD Mutations. *Cell* (2008). doi:10.1016/j.cell.2008.04.030

4. Hyeon, C. & Thirumalai, D. Capturing the essence of folding and functions of biomolecules using coarse-grained models. *Nat Commun* **2,** 487 (2011).

5. Noel, J. & Onuchic, J. in *Computational Modeling of Biological Systems* (ed. Dokholyan, N. V) 31–54 (Springer US, 2012). doi:10.1007/978-1-4614-2146-7_2

6. Pincus, D. L., Cho, S. S., Hyeon, C. & Thirumalai, D. Minimal models for proteins and RNA from folding to function. *Prog Mol Biol Transl Sci* **84,** 203–250 (2008).

7. Whitford, P. C., Sanbonmatsu, K. Y. & Onuchic, J. N. Biomolecular dynamics: order-disorder transitions and energy landscapes. *Rep Prog Phys* **75,** 76601 (2012).

8. Bottaro, S., Di Palma, F. & Bussi, G. The role of nucleobase interactions in RNA structure and dynamics. *Nucleic Acids Res.* **42,** 13306–13314 (2014).

9. Whitford, P. C. *et al.* Nonlocal helix formation is key to understanding S-adenosylmethionine-1 riboswitch function. *Biophys. J.* **96,** L7--L9 (2009).

10. Whitford, P. C. *et al.* An all-atom structure-based potential for proteins: bridging minimal models with all-atom empirical forcefields. *Proteins* **75,** 430–441 (2009).

11. Noel, J. K., Whitford, P. C., Sanbonmatsu, K. Y. & Onuchic, J. N. SMOG@ctbp: simplified deployment of structure-based models in GROMACS. *Nucleic Acids Res.* **38,** W657--W661 (2010).

12. Macke, T. J. & Case, D. A. in *Molecular Modeling of Nucleic Acids* 379–393 (1998). doi:10.1021/bk-1998-0682.ch024

13. Wong, K.-Y. & Pettitt, B. M. The pathway of oligomeric DNA melting investigated by molecular dynamics simulations. *Biophys. J.* **95,** 5618–5626 (2008).

14. Perez, A. & Orozco, M. Real-time atomistic description of DNA unfolding. *Angew. Chem. Int. Ed. Engl.* **49,** 4805–4808 (2010).

15. Hagan, M. F., Dinner, A. R., Chandler, D. & Chakraborty, A. K. Atomistic understanding of kinetic pathways for single base-pair binding and unbinding in DNA. *Proc. Natl. Acad. Sci. U. S. A.* **100,** 13922–13927 (2003).

16. Deng, N.-J. & Cieplak, P. Free energy profile of RNA hairpins: a molecular dynamics simulation study. *Biophys. J.* **98,** 627–636 (2010).

17. Wang, Y., Wang, Z., Wang, Y., Liu, T. & Zhang, W. The nearest neighbor and next nearest neighbor effects on the thermodynamic and kinetic properties of RNA base pair. *J. Chem. Phys.* (2018). doi:10.1063/1.5013282

18. Colizzi, F. & Bussi, G. RNA unwinding from reweighted pulling simulations. *J. Am. Chem. Soc.* **134,** 5173–5179 (2012).

19. Cheatham III, T. E. & Kollman, P. A. MOLECULAR DYNAMICS SIMULATION OF NUCLEIC ACIDS. *Annu. Rev. Phys. Chem.* **51,** 435–471 (2000).

20. Cieplak, P., Cheatham, T. E. & Kollman, P. A. Molecular Dynamics Simulations Find That 3’ Phosphoramidate Modified DNA Duplexes Undergo a B to A Transition and Normal DNA Duplexes an A to B Transition. *J. Am. Chem. Soc.* **119,** 6722–6730 (1997).

21. Liu, J. D., Zhao, L. & Xia, T. The dynamic structural basis of differential enhancement of conformational stability by 5'- and 3'-dangling ends in RNA. *Biochemistry* **47,** 5962–5975 (2008).

22. Mohan, S. *et al.* Mechanism of RNA double helix-propagation at atomic resolution. *J. Phys. Chem. B* **113,** 2614–2623 (2009).

23. Sugimoto, N., Kierzek, R. & Turner, D. H. Sequence dependence for the energetics of dangling ends and terminal base pairs in ribonucleic acid. *Biochemistry* **26,** 4554–4558 (1987).

24. Bommarito, S., Peyret, N. & SantaLucia Jr, J. Thermodynamic parameters for DNA sequences with dangling ends. *Nucleic Acids Res.* **28,** 1929–1934 (2000).

25. Isaksson, J. & Chattopadhyaya, J. A uniform mechanism correlating dangling-end stabilization and stacking geometry. *Biochemistry* **44,** 5390–5401 (2005).

26. Arunajadai, S. G. RNA unwinding by NS3 helicase: a statistical approach. *PLoS One* **4,** e6937 (2009).

27. Dumont, S. *et al.* RNA translocation and unwinding mechanism of HCV NS3 helicase and its coordination by ATP. *Nature* **439,** 105 (2006).

28. Cheng, W., Dumont, S., Tinoco Jr, I. & Bustamante, C. NS3 helicase actively separates RNA strands and senses sequence barriers ahead of the opening fork. *Proc. Natl. Acad. Sci. U. S. A.* **104,** 13954–13959 (2007).

29. Fairman-Williams, M. E., Guenther, U.-P. & Jankowsky, E. SF1 and SF2 helicases: family matters. *Curr. Opin. Struct. Biol.* **20,** 313–324 (2010).

30. Pyle, A. M. Translocation and Unwinding Mechanisms of RNA and DNA Helicases. *Annu. Rev. Biophys.* **37,** 317–336 (2008).

31. Singleton, M. R., Dillingham, M. S. & Wigley, D. B. Structure and mechanism of helicases and nucleic acid translocases. *Annu. Rev. Biochem.* **76,** 23–50 (2007).

32. Pyle, A. M. RNA helicases and remodeling proteins. *Curr. Opin. Chem. Biol.* **15,** 636–642 (2011).

33. Byrd, A. K. & Raney, K. D. Superfamily 2 helicases. *Front Biosci (Landmark Ed)* **17,** 2070–2088 (2012).

34. Ding, S. C. & Pyle, A. M. Molecular mechanics of RNA translocases. *Methods Enzym.* **511,** 131–147 (2012).

35. He, Y., Andersen, G. R. & Nielsen, K. H. The function and architecture of DEAH/RHA helicases. *Biomol. Concepts* **2,** 315–326 (2011).

36. Raney, K. D., Byrd, A. K. & Aarattuthodiyil, S. Structure and Mechanisms of SF1 DNA Helicases. *Adv. Exp. Med. Biol.* **973,** E1 (2013).

37. Chakrabarti, S. *et al.* Molecular mechanisms for the RNA-dependent ATPase activity of Upf1 and its regulation by Upf2. *Mol. Cell* **41,** 693–703 (2011).

38. Adedeji, A. O. *et al.* Mechanism of nucleic acid unwinding by SARS-CoV helicase. *PLoS One* **7,** e36521 (2012).

39. Karpe, Y. A. & Lole, K. S. NTPase and 5' to 3' RNA duplex-unwinding activities of the hepatitis E virus helicase domain. *J. Virol.* **84,** 3595–3602 (2010).

40. Shu, Z., Vijayakumar, S., Chen, C.-F., Chen, P.-L. & Lee, W.-H. Purified human SUV3p exhibits multiple-substrate unwinding activity upon conformational

change. *Biochemistry* **43,** 4781–4790 (2004).

41. Wang, D. D.-H., Shu, Z., Lieser, S. A., Chen, P.-L. & Lee, W.-H. Human mitochondrial SUV3 and polynucleotide phosphorylase form a 330-kDa heteropentamer to cooperatively degrade double-stranded RNA with a 3'-to-5' directionality. *J. Biol. Chem.* **284,** 20812–20821 (2009).

42. Bar-Ziv, R. & Libchaber, a. Effects of DNA sequence and structure on binding of RecA to single-stranded DNA. *Proc. Natl. Acad. Sci. U. S. A.* (2001). doi:10.1073/pnas.151242898

43. Jezewska, M. J. & Bujalowski, W. Interactions of Escherichia coli replicative helicase PriA protein with single-stranded DNA. *Biochemistry* (2000). doi:10.1021/bi001113y

44. Dehghani-Tafti, S. & Sanders, C. M. DNA substrate recognition and processing by the full-length human UPF1 helicase. *Nucleic Acids Res.* (2017). doi:10.1093/nar/gkx478

45. Khaki, A. R. *et al.* The Macroscopic Rate of Nucleic Acid Translocation by Hepatitis C Virus Helicase NS3h Is Dependent on Both Sugar and Base Moieties. *J. Mol. Biol.* (2010). doi:10.1016/j.jmb.2010.04.065

46. Tomko, E. J. & Lohman, T. M. Modulation of Escherichia coli UvrD Single-Stranded DNA Translocation by DNA Base Composition. *Biophys. J.* (2017). doi:10.1016/j.bpj.2017.08.023
